# Supplementary material for: Studying trajectories of multimorbidity: a systematic scoping review of longitudinal approaches and evidence
Source: BMJ Open. 2021 Nov 22;11(11):e048485. doi: 10.1136/bmjopen-2020-048485 (PMC8609933; doi:10.1136/bmjopen-2020-048485)
Supplement: Author's manuscript [file bmjopen-2020-048485.draft_revisions.pdf]

BMJ Open

BMJ Open is committed to open peer review. As part of this commitment we make the peer review history of every article we publish publicly available.

When an article is published we post the peer reviewers' comments and the authors' responses online. We also post the versions of the paper that were used during peer review. These are the versions that the peer review comments apply to.

The versions of the paper that follow are the versions that were submitted during the peer review process. They are not the versions of record or the final published versions. They should not be cited or distributed as the published version of this manuscript.

BMJ Open is an open access journal and the full, final, typeset and author-corrected version of record of the manuscript is available on our site with no access controls, subscription charges or pay-per-view fees (http://bmjopen.bmj.com).

If you have any questions on BMJ Open's open peer review process please email info.bmjopen@bmj.com

BMJ Open

Studying trajectories of multimorbidity: a systematic scoping review of longitudinal approaches and evidence

Journal:	*BMJ Open*
Manuscript ID	bmjopen-2020-048485
Article Type:	Original research
Date Submitted by the Author:	29-Dec-2020
Complete List of Authors:	Cezard, Genevieve; University of St Andrews, Geography and Sustainable Development<br>McHale, C; University of St Andrews, School of Medicine<br>Sullivan, Frank; University of St Andrews, School of Medicine<br>Bowles, Juliana; University of St Andrews, Computer Science<br>Keenan, Katherine; University of St Andrews, Geography and Sustainable Development
Keywords:	PREVENTIVE MEDICINE, PUBLIC HEALTH, INTERNAL MEDICINE, EPIDEMIOLOGY

SCHOLARONE™
Manuscripts

I, the Submitting Author has the right to grant and does grant on behalf of all authors of the Work (as defined in the below author licence), an exclusive licence and/or a non-exclusive licence for contributions from authors who are: i) UK Crown employees; ii) where BMJ has agreed a CC-BY licence shall apply, and/or iii) in accordance with the terms applicable for US Federal Government officers or employees acting as part of their official duties; on a worldwide, perpetual, irrevocable, royalty-free basis to BMJ Publishing Group Ltd ("BMJ") its licensees and where the relevant Journal is co-owned by BMJ to the co-owners of the Journal, to publish the Work in this journal and any other BMJ products and to exploit all rights, as set out in our licence.

The Submitting Author accepts and understands that any supply made under these terms is made by BMJ to the Submitting Author unless you are acting as an employee on behalf of your employer or a postgraduate student of an affiliated institution which is paying any applicable article publishing charge ("APC") for Open Access articles. Where the Submitting Author wishes to make the Work available on an Open Access basis (and intends to pay the relevant APC), the terms of reuse of such Open Access shall be governed by a Creative Commons licence – details of these licences and which Creative Commons licence will apply to this Work are set out in our licence referred to above.

Other than as permitted in any relevant BMJ Author's Self Archiving Policies, I confirm this Work has not been accepted for publication elsewhere, is not being considered for publication elsewhere and does not duplicate material already published. I confirm all authors consent to publication of this Work and authorise the granting of this licence.

For peer review only

Studying trajectories of multimorbidity: a systematic scoping review of longitudinal approaches and evidence.

Genevieve Cezard [1], Calum McHale [2], Frank Sullivan [2], Juliana Bowles [3], Katherine Keenan [1*]

0

[1] School of Geography and Sustainable Development, University of St Andrews, St Andrews, UK.

[2] School of Medicine, University of St Andrews, St Andrews, UK.

[3] School of Computer Science, University of St Andrews, St Andrews, UK.

*Corresponding author: Dr Katherine Keenan, School of Geography and Sustainable Development, University of St Andrews, Irvine Building, North Street, St Andrews, UK. KY16 8AL. Katherine.keenan@st-andrews.ac.uk

Word count: 4,604.

For peer review only

ABSTRACT

Objectives: Multimorbidity – the co-occurrence of at least two chronic diseases in an individual- is an important public health challenge in ageing societies. The vast majority of multimorbidity research takes a cross-sectional approach, but longitudinal approaches to understanding multimorbidity are an emerging research area, being encouraged by multiple funders. To support development in this research area, the aim of this study is to scope the methodological approaches and substantive findings of studies which have investigated longitudinal multimorbidity trajectories.

Design: We conducted a systematic search for relevant studies in four online databases (Medline, Scopus, Web of Science, and Embase) using pre-defined search terms and inclusion and exclusion criteria. The search was complemented by searching reference lists of relevant papers. From the selected studies we systematically extracted data on study methodology and findings, and summarised them in a narrative synthesis.

Results: We identified 34 studies investigating multimorbidity longitudinally, all published in the last decade, and predominantly in high-income countries from the Global North. Longitudinal approaches employed included constructing change variables, multilevel regression analysis (e.g. growth curve modelling), longitudinal group-based methodologies (e.g. latent class modelling), analysing disease transitions, and visualisation techniques. Commonly identified risk factors for multimorbidity onset and progression were older age, higher socio-economic and area-level deprivation, overweight, and poorer health behaviours.

Conclusion: The nascent research area employs a diverse range of longitudinal approaches that characterize accumulation and disease combinations, and to a lesser extent disease sequencing and progression. Gaps include understanding the long-term, life course determinants of different multimorbidity trajectories, and doing so in across diverse populations, including those from low and middle-income countries. This can provide a detailed picture of morbidity development, with important implications from a clinical and intervention perspective.

Keywords: Multimorbidity; longitudinal; cohort; trajectories; systematic review.

For peer review only - http://bmjopen.bmj.com/site/about/guidelines.xhtml

For peer review only

STRENGTHS AND LIMITATIONS OF THE STUDY

- This is the first systematic review to focus on studies that take a longitudinal, rather than cross-sectional, approach to multimorbidity.
- Systematic searches of online academic databases were performed using pre-defined search terms, as well as searching of reference lists, and this is reported using PRISMA guidelines.
- For selected papers, data was double extracted using standardised proformas to aid narrative synthesis.
- Due to the heterogeneity of the studies included, their weaknesses were described in the narrative synthesis, but we did not perform quality assessment using standardised tools.

For peer review only

For peer review only - http://bmjopen.bmj.com/site/about/guidelines.xhtml

INTRODUCTION

The term multimorbidity is used to define the co-occurrence of multiple diseases, specifically two or more chronic conditions within the same individual [1,2]. Multimorbidity represents a huge immediate and future challenge for health care systems around the world. The global prevalence of multimorbidity is expected to increase through the 21st century, as a result of increased life expectancy, population ageing, and the expansion of morbidity. For example, within the United Kingdom, the prevalence of 'complex multimorbidity'- defined as four or more co-occurring chronic conditions- is projected to double within the next 15 years[3]. The implications of this for individuals and societies are stark: multimorbidity is predictive of poorer quality of life [4], greater functional decline [5], and increased mortality [6]. Management and treatment of multimorbidity also places a considerable economic and logistical burden on health services [7], which are not adapted to deal with multimorbidity, being typically organised around the single disease model.

In response to this challenge, in the last two decades there has been an explosion of (predominantly cross-sectional) research that has investigated the risk factors and patterns of multimorbidity. For example, systematic reviews have identified common clusters of diseases[8–10], which include cardiovascular and metabolic diseases, mental health conditions, and musculoskeletal disorders. Key risk factors for the multimorbidity development and severity include increasing age and poor socioeconomic status (SES)[10,11], and poor health behaviours, such as high body mass index (BMI) and smoking[12]. However, the vast majority of multimorbidity studies apply a cross-sectional approach; longitudinal approaches are scarce. To date there are more than 70 published systematic reviews about multimorbidity, covering definitions to interventions (for example[2,13]), and none of these focuses on reviewing longitudinal studies. While 'snap-shot' analyses are useful for understanding prevalence and clustering of diseases, they provide little information on multimorbidity development over time and sequencing of diseases, which have important implications from a clinical and intervention perspective. Recently, there has been a growing orientation towards longitudinal approaches by academic communities and funders such as the UK's Academy of Medical Science [14]. This paper aims to systematically review the emerging body of literature of longitudinal studies that have investigated multimorbidity trajectories. Our research questions are:

1. What type and range of longitudinal methods are used to analyse multimorbidity over time within individuals?

For peer review only

2. What are the risk/protective factors identified to be associated with individual multimorbidity trajectories?

We systematically review the literature via a scoping review approach rather than a systematic review approach[15]. In reporting, we follow the recently developed Preferred Reporting Items for Systematic Reviews and Meta-Analyses for scoping reviews (PRISMA-ScR) [16].

METHODS

Eligibility criteria

Inclusion and exclusion criterion were defined prior to database searches. A primary eligibility criterion was to measure multimorbidity longitudinally within the same sample of adults using a quantitative approach; and we excluded cross-sectional or qualitative designs, reviews, meta-analyses and commentary that did not contain empirical results. Studies had to measure multimorbidity through recognised diseases /conditions or a defined multimorbidity measure such as the Charlson or Elixhauser comorbidity indices [17,18], but not solely a collection of symptoms/states (such as disability or frailty) or disease risk factors (such as obesity). Studies were required to measure change in multimorbidity between distinguishable diseases rather than progression within a single disease category (e.g. different types of cancer). We also excluded studies that examined transitions from an index disease into a secondary disease (e.g. comorbidities of diabetes). Finally, included studies were focussed on adult humans (aged 18+), and were peer-reviewed journal articles, written in English language.

Search strategy

Four online databases were searched: Medline, Scopus, Web of Science, and Embase. Initially, scoping searches were conducted within each database, with relevant terms such as ‘multimorbidity’, ‘disease trajectory’, and ‘longitudinal’, in order to develop and refine the final search strategy (Table 1).

For peer review only - http://bmjopen.bmj.com/site/about/guidelines.xhtml

Table 1. Summary of search strategy

Search no.	Search terms
#1	Multimorbidity (multimorbid*; multi-morbid*) OR Comorbidity (comorbid*; co-morbidi*) OR Cooccurrence (cooccur*; co-occur*)
#2	disease OR condition OR illness AND cluster* OR trajectory (trajector*) OR cascade* OR accumulation (accumulat*) OR combination* OR sequence (sequenc*) OR transition*
#3	cohort* OR longitudinal* OR prospective*
#4	#1 AND #2 AND #3
#5	#4 AND NOT cell* OR gene OR genes OR bacteria* OR DNA OR COVID-19 (covid*)

The final search was a combination of three search elements: first, the concept of multimorbidity, second the methodological approach of disease trajectories, and third, longitudinal study design. These search terms initially returned a large number of irrelevant references, focusing on cellular medicine, genetics, and Covid-19, so we added an additional condition to exclude these. We also refined the search results to include English language, adult humans, and peer-reviewed journal articles only. All searches were conducted in May 2020 and no date restrictions were used. The full search syntax is included in Appendix A. We identified additional relevant papers through recommendations from co-authors and external collaborators. The database search results were searched for these additional papers, and if they were not identified in the database searches, they were included as *'identified through other sources'* and were subject to the same screening procedure as papers identified through database searches.

Screening and study selection

After de-duplication, articles were screened for eligibility by title, abstract and finally full text (work shared between GC, CM, and KK). At full text stage, a double screening process was used to minimise evidence selection bias[19], meaning two co-authors blindly and independently reviewed the study for inclusion. Any disagreements were resolved through discussion and consensus. The reference lists of the selected studies were screened to identify any relevant studies that may have been missed in the main search, and any newly identified articles were subject to the same screening and data extraction processes.

For peer review only - http://bmjopen.bmj.com/site/about/guidelines.xhtml

Data extraction and synthesis

Three authors (GC, CM, KK) extracted and double-extracted information from selected articles. We extracted information on study and sample characteristics, including the title, authors and publication year, study setting, data source used, information on the study population (e.g. inclusion and exclusion criteria, sample size and age), and follow-up duration. We also extracted study objectives, information on multimorbidity conceptualisation and measures, and methodological and analytical approaches, focussing on those specifically used for the analysis of multimorbidity trajectories. Finally, we extracted the key findings, and limitations reported in each study in relation to generalisability, accuracy, comprehensiveness, methodology and interpretation.

To develop the narrative synthesis, we analysed and summarised the patterns in the extracted data, investigated the similarities and differences between studies, and examined bias and limitations to identify knowledge gaps and the strengths and weaknesses of methodological approaches.

Patient and Public Involvement

No patient involved.

RESULTS

Study selection

Figure 1 depicts the study selection process. Database searches returned 11,420 articles and 9 additional papers were identified from other sources. Of the combined 11,429 papers, 4,705 were duplicate references and removed. Of the remaining 6,724 papers, 6,315 were removed during title screening and a further 361 papers during abstract screening. The most common reasons for exclusion were studies which did not focus on *multimorbidity* longitudinally (e.g. trajectories were followed within a single disease) and study design not being longitudinal (e.g. cross-sectional analysis). The remaining 48 papers went through full-text screening and 19 subsequently were removed during this process. Searching the reference lists of the remaining 29 papers identified another 11 potentially relevant papers. After screening these 11 papers, 6 were excluded leaving 5 additional papers for inclusion. In total, 34 papers were selected for further data extraction.

[Fig 1 about here]

For peer review only - http://bmjopen.bmj.com/site/about/guidelines.xhtml

Characteristics of the selected studies

Table 2 summarises the study characteristics. All articles were published since 2011, and were primarily based data from European countries (N=15)[5,20–33], Australia and North America (N=13)[34–46], and high-income Asian countries Taiwan and Singapore (N=3)[47–49]. Apart from one study using Chinese data[50], none related to low- and middle-income settings.

Sample characteristics varied widely. For example, sample size ranged from 756 in a survey of older participants[39] to 6.2 million in a nationwide study using Danish register data[21], and the length of follow-up periods ranged between 2 and 20 years. Most studies had age restrictions, with about half focused on older populations (50 years+), and one study focused on the very old (80 years plus)[32]. Most samples included both males and females apart from two studies including males only [23,33] and three studies including females only [37,41,45]. Furthermore, three studies focused on U.S. veterans, a predominantly male population [34,42,51]. The data sources used were a combination of administrative data, including primary or secondary care records, disease registries, and health insurance data (23 studies), and survey data (17 studies). There were common datasets used across studies, such as the Swedish National study on Aging and Care in Kungsholmen (SNAC-K) [24,27–29]. In six studies, survey data was combined with administrative data sources [24,25,27–29,38]. In five survey-based studies, questionnaire data was supplemented by medical examination records, or cognitive or laboratory tests [30,39,50,52,53]. Informed consent of participants was mentioned in 10 of the 17 studies using survey data.

For peer review only

For peer review only - http://bmjopen.bmj.com/site/about/guidelines.xhtml

Table 2: Characteristics of the selected studies

Study, year	Country	Sample size	Baseline sample age	Follow-up period	Data source	Number of conditions included	Disease identification strategy
Alaeddini et al. (2017)	USA	N=601,805	Adults	14 years	Administrative data: inpatient and outpatient data	4	ICD-9-CM codes
Ashworth et al. (2019)	UK	N=332,353	18 years+	14.5 years	Administrative data: primary care records	12	Read codes in UK primary care (based on the Quality and Outcome Framework)
Beck et al. (2016)	Denmark	N≈120,000	All ages	18.5 years	Administrative data: inpatient and outpatient data, and emergency visits	All diseases taken at three level ICD 10 code	ICD 10 codes (A41 specified for sepsis)
Calderon-Larranaga et al. (2018)	Sweden	N=2,387	60 years+	3 to 6 years	Survey with interviews, clinical examinations, cognitive tests, and laboratory tests combined with and administrative data: National Patient Register, death	918 diseases grouped into 60 categories	ICD10 codes
Calderon-Larranaga et al. (2019)	Sweden	N=2,293	60 years+	9 years	Survey with interviews, clinical examinations, cognitive tests, and laboratory tests combined with and administrative data: National Patient Register, death	918 diseases grouped into 60 categories	ICD10 codes
Canizares et al. (2017)	Canada	N=10,186	20 -69 years	18 years	Survey data	17	Self-reported
Chang et al. (2011)	Taiwan	N=147,892	All ages	3 years	Administrative data: national insurance claims data including inpatient, outpatient, ambulatory care, dental care, and pharmacy expenditure	Not specified	ICD-9-CM codes
Dekhtyar et al. (2019)	Sweden	N=2,589	60 years+	9 years	Survey with interviews, clinical examinations, cognitive tests, and laboratory tests combined with and administrative data: National Patient Register, death	918 diseases grouped into 60 categories	ICD10 codes
Fabbri et al. (2015)	Italy	N=1,018	60 years+	9 years	Survey data with clinical examinations and laboratory tests	15	Self-reported medical history, medication use, medical documentation, and clinical examination.
Fabbri et al. (2016)	USA	N=756	65 years+	9 years	Survey data with clinical examinations and laboratory tests	13	Self-reported, laboratory tests, and clinical examination
Faruqui et al. (2018)	USA	N=257,633	18 years+	5 years	Administrative data: inpatient and outpatient data	5	ICD-9-CM codes
Fraccaro et al. (2016)	UK	N=287,459	18 years+	9.5 years	Administrative data: primary and secondary care, death, and out-migration data	22	Read codes in UK primary care data (based on validated codes by Khan et al. (2010))
Gellert et al. (2018)	Germany	N=1,398	80 years+	6 years	Administrative data: ambulatory, hospital, and long-term care	30	ICD10 codes
Hanson et al. (2015)	USA	N=41,158	65-84 years	18 years	Administrative data : UPDB, death, and Medicare claims	17	Diseases are identified following the SEER-Medicare Comorbidity macro from Medicare data in 1992-2009
Hiyoshi et al. (2017)	Sweden	N=5,218	44-51 years	5 years	Administrative data: Cancer register and Patient register with inpatient and outpatient data	18	Swedish version of ICD-9 and 10
Hsu (2015)	Taiwan	N=2,584	60 years+	14 years	Survey data	15 conditions grouped into 6 disease types	Self-reported
Jackson et al. (2015)	Australia	N=4,865	47-52 years	12 years	Survey data	18	Self-reported
Jensen et al. (2014)	Denmark	N=6.2 million	All ages	14.9 years	Administrative data: inpatient, outpatient, and emergency visits	All diseases taken at three level ICD 10 code	ICD10 codes
Kim et al. (2018)	South Korea	N=121,733	60 years+	11 years	Administrative data: claims data from private healthcare providers.	20	Claims data

For peer review only - http://bmjopen.bmj.com/site/about/guidelines.xhtml

Lappenschaar et al. (2013)	The Netherlands	N=182,396	35 years+	9 years	Administrative data: primary care data.	6	International Classification of Primary Care (ICPC) codes, completed with laboratory tests and medication information.
Lindhagen et al. (2015)	Sweden	N=20,237	Adults	10 years	Administrative data: cancer and population registers.	17	ICD codes from Patient and Cancer Registries
Perez et al. (2020)	Sweden	N=2,596	60 years+	6 years	Survey with interviews, clinical examinations, cognitive tests, and laboratory tests combined with and administrative data: National Patient Register, death	918 diseases grouped into 60 categories	ICD10 codes
Pugh et al. (2016)	USA	N=164,933 (derivation) N=146,051 (validation)	Adults	3 years	Administrative data: inpatient, outpatient, and medications data	Not specified	ICD-9-CM codes
Quinones et al. (2011)	USA	N=17,517	51 years+	11 years	Survey data	7	Self-reported
Quinones et al. (2019)	USA	N=8,331	51- 55 years	16 years	Survey data	7	Self-reported
Rocca et al. (2016)	USA	N=3,306	Adults < 50	10-19 years	Administrative data: medical records.	18	ICD-9 codes
Ruel et al. (2014)	Australia	N=1,854	18 years+	6-10 years	Survey (with telephone interview, self-completed questionnaire, and clinic biomedical assessment including blood sample)	8	Self-reported and biomedical measurements
Ruel et al. (2014)	China	N=1,020	20 years+	5 years	Survey (with laboratory tests)	11	Self-reported, blood samples, and clinical examinations
Ryan et al. (2018)	Republic of Ireland	N=4,502	50 years+	2 years	Survey (with health assessment in a health centre)	30 diseases grouped into 16 categories	Self-reported and clinical examination
Siriwardhana et al. (2018)	USA (Hawaii)	N=22,930	65 years+	5 years	Administrative data (health insurance data)	3	ICD9 codes
Strauss et al. (2014)	UK	N=24,615 (Phase I) N=4,532 (Phase II)	50 years+	3 years	Administrative data (primary care records for CiPCA) linked to Questionnaire data (NorStOP)	42	Read codes in UK primary care
Xu et al. (2018)	Australia	N=11,941	45-50 years	20 years	Survey data (self-reported)	3	Self-reported
Zeng et al. (2014)	USA (Colorado)	N=961 (Primary cohort) N=13,163 (secondary cohort)	65 years+	10 years	Administrative data (electronic health records including inpatient admission and emergency visits, and death) and survey	17	ICD-9-CM codes based on Quan et al. (2005) algorithm
Zhu et al. (2018)	Singapore	N≈700,000	All ages	7 years	Administrative data (electronic health records and deaths)	6	Not specified

For peer review only - http://bmjopen.bmj.com/site/about/guidelines.xhtml
For peer review only

Methods of disease and multimorbidity ascertainment

Studies based on administrative data relied on clinician-diagnosed diseases, often using standardised diagnosis codes, such as the International Classification of Diseases i.e. ICD-9, ICD-9-CM, or ICD-10. All survey-based studies used participant self-report for disease identification. Studies that combined survey data with other sources ascertained disease status mostly through clinician-diagnosis [24,25,27–29,38] but some supplemented with laboratory and cognitive tests[30,39,50,52,53]. The number of diseases which were considered to contribute to the measure of multimorbidity varied widely, ranging from three [36,37] to a very large number based on three levels of ICD-10 codes [21,26]. Studies using survey data used a narrower range of diseases than those drawn from administrative data. The precise list of diseases was never uniform between studies (see Appendix B for full details), but the rationale for choosing them was usually described. For example, included diseases with high prevalence and risk of disability and mortality [30,39], or that were assessed/validated by clinicians [24,25,27–29]. Some used lists based on the Charlson and Elixhauser multimorbidity indices [23,31–33,38,40,54] but these were sometimes augmenting with extra conditions [33] or reduced due to data sensitivity restrictions[31].

Approaches to the measurement of multimorbidity trajectories

To develop longitudinal measures of multimorbidity, studies tended to take one of two broad approaches. The most common was that repeated measures of multimorbidity status over time were measured for each individual. This mainly involved constructing unweighted or weighted counts of diseases at regular intervals for each individual, thus conceptualising multimorbidity as a continuum (for example [50,52]), although a few still used a binary measure of two or more chronic conditions[35,53]. The second broad approach explored disease sequences or disease transitions [21,22,26,34,34,36,37,49]. Only one study explored the order of disease occurrence[20]. Finally, one study took both approaches of using a multimorbidity count and assessing disease transitions[37].

Breaking this down further, we identified five broad analytical approaches: constructed variables of multimorbidity change, multilevel regression modelling, transition and data mining methodologies, visual approaches (articles summarised in Table 3) and longitudinal group-based methodologies (articles summarised in Table 4). Note that some studies employed more than one type of approach (e.g. Xu 2018 [37]). In the first approach, four articles created variables of multimorbidity change[31,47,50,53]. In one study, intra-individual change in Charlson Comorbidity Index (CCI) between baseline and later time points was used [31], and in another, transitions to two or more conditions, or by acquisition of additional conditions[53]. Two studies used simple methods to

For peer review only - http://bmjopen.bmj.com/site/about/guidelines.xhtml

construct morbidity trajectory groups (e.g. 'constant high', 'constant medium', 'constant low',)[47] and disease transition stages (e.g. 'healthy', 'healthy to a single chronic disease')[50]. After creating these categorical dependent variables the authors used them in regression analysis to assess their association with health expenditures[47], diet[50], and physical activity and functioning[53].

The next approach, employed by 14 studies (Table 3) [24,27–32,35,37–39,43,44,52] was multilevel regression modelling (variously referred to as random effects models, growth curve models, hierarchical linear models or multilevel models). These studies analyse repeated measures of multimorbidity within each individual, considering this as a 'trajectory' or 'growth curve'. The dependent variable was typically a count of diseases or a multimorbidity index measured repeatedly, and the coefficients assessed a change in this over time, many including random effects for both the intercept and slope. One study used the regression estimates (i.e. intercept and slope coefficients for multimorbidity) to create categories capturing the pace of multimorbidity e.g. 'rapidly accumulating' and 'slowly accumulating', which were used for further modelling[27]. Another study used logistic regression generalized estimating equations to model transitions between fixed disease states[37]. Many of these studies investigated whether certain covariates such as biomarkers[24,30], socio-demographics and life experiences [29] affected the pace of change in multimorbidity by including an interaction term between time and the respective covariates.

For peer review only

Table 3. Methods of studies taking four analytical approaches (multimorbidity change variables, regression, transition modelling and visual approaches)

Study, year	Main study outcome	Statistical methodology used to analyse multimorbidity longitudinally
**Approach 1: Constructed variables of multimorbidity change (N=4)**		
Chang et al. (2011)	Prospective medical use and expenditures	**Morbidity trajectory groups**: constant high, constant medium, constant low, decreasing, increasing, and erratic.
Fraccaro et al. (2016)	Time to death from any cause	**Change in comorbidities:** difference between baseline Charlson Comorbidity Index (CCI) and 1, 5, 10-year follow-up CCI scores as a proportion of mortality rate.
Ruel et al. (2014)	Six chronic disease transition stages groups	**Groups of chronic disease transition stages** (healthy, healthy to a single chronic disease, stable with a single chronic disease, healthy to multimorbidity, stable multimorbidity, and increasing multimorbidity)
Ryan et al. (2018)	Development/worsening of multimorbidity	**Constructed measures of multimorbidity change** (from zero or one condition at baseline to two or more conditions) and **of worsening of multimorbidity** (from multimorbidity at baseline to additional conditions at follow-up)
**Approach 2: Regression-based approaches (N=14)**		
Calderon-Larranaga et al. (2018)	Disability (IADL[1] count)	**Linear mixed models[2]** estimated the rate of multimorbidity accumulation. Predicted slopes are examined by quartile and further dichotomised into "rapidly accumulating" (upper quartile) and "slowly accumulating" (three lower quartile).
Calderon-Larranaga et al. (2019)	Level of multimorbidity (count of chronic conditions) and disability (IADL count)	**Linear mixed models** assessed the association between baseline level of psychological factors and multimorbidity and disability over time. The interaction terms between time and each of the psychological factors were included as a fixed effect.
Canizares et al. (2017)	Multimorbidity (binary- 2 or more conditions)	**Multilevel logistic growth modelling** was used to examine the age, period, and cohort effects on multimorbidity. Observations nested in individuals and age and birth cohort entered as fixed effects.
Dekhtyar et al. (2019)	Level of multimorbidity (count of chronic conditions)	**Linear mixed models** assessed the association between life experiences and the speed of multimorbidity accumulation over time. Interaction terms between time and life experiences included as fixed effects.
Fabbri et al. (2015)	Level of multimorbidity (count of chronic conditions)	**Linear mixed models** assessed the association between baseline age, disease status, and biomarkers with the number of diseases over follow-up. The study also tested whether increased IL-6 over time would predict steeper increase in multimorbidity over time, independent of baseline IL-6.
Fabbri et al. (2016)	Standardized neurocognitive tests evaluating cognitive function	**Linear mixed models** were used to estimate rate of change in multimorbidity (count of diseases). The individual slopes of multimorbidity rise were dichotomized into faster accumulation (upper quartile) and the rest (lower 3 quartiles).
Fraccaro et al. (2016)	All-cause mortality	Multimorbidity change was measured by differences between baseline Charlson Comorbidity Index (CCI) and 1, 5, 10-year follow-up CCI scores as a proportion of mortality rate. **Survival analysis (Cox regression)** estimated mortality rates as a function of age, gender and CCI scores (fixed and time-varying).
Gellert et al. (2018)	Level of multimorbidity (count of comorbidities- based on Elixhauser)	**Linear mixed models** (random intercepts and slopes) estimated differential increase in the number of comorbidities over 25 calendar quarters prior to death in centenarian, nonagenarian, and octogenarian cohorts.
Perez et al. (2020)	Level of multimorbidity (count of chronic conditions)	**Linear mixed models** (random intercepts and slopes) were employed to analyse the association between baseline tGSH levels and level of multimorbidity.
Quinones et al. (2011)	Level of multimorbidity (count of chronic conditions)	**Linear mixed models** (random intercepts and slopes) analysed ethnic variations in level of multimorbidity.
Quinones et al. (2019)	Level of multimorbidity (count of chronic conditions)	**Negative binomial generalized estimating equation (GEE) models** with a first-order autoregressive covariance structure were used to assess the relationship between chronic disease accumulation and race/ethnicity.
Ruel et al. (2014)	Count of chronic conditions + incidence of multimorbidity	**Multinomial logistic regression** estimated the count and individual proportion of chronic diseases in those with no or one chronic disease at baseline.

For peer review only - http://bmjopen.bmj.com/site/about/guidelines.xhtml

Xu et al. (2018)	Cumulative incidence of three conditions (diabetes, heart disease and stroke)	**Repeated measures logistic regression** using **generalized estimating equations (GEE)** were used to identify risk factors for developing three conditions and their combinations.
Zeng et al. (2014)	Self-reported health, number of primary care visits, inpatient admissions, emergency department visits, and mortality	**Linear mixed models** (random intercepts and slopes) estimated the individual trajectory of Charlson Comorbidity Index (CCI) over time (up to 10 years), which was used as an independent variable in a subsequent linear regression model for the health outcomes.
**Approach 3: Transition and disease progression modelling (N=8)**		
Alaeddini et al. (2017)	Clusters of disease transition considering four conditions (hypertension, depression, PTSD and back pain)	Disease transitions were modelled using **Markov chain models with a transition matrix, placed in a Latent Regression Markov Mixture Model** to incorporate subject–specific covariates (e.g., age, sex, race/ethnicity, etc.). Markov Clustering algorithm used to identify patterns of disease progression.
Beck et al. (2016)	30-day mortality in patients with sepsis	Data driven method combining **temporal directed pairs** for identification of disease trajectories based on the method developed by Jensen (2014)
Faruqui et al. (2018)	Development of 5 specific conditions	Comparison of several methods: **unsupervised Bayesian network, multivariate regression and latent regression Markov mixture modelling. Longest Path Algorithm (LPA)** from the Bayesian network was used to identify the most probable sequence from/to a specific disease.
Jensen et al. (2014)	Disease trajectories	**Temporal correlation analysis** i.e. strength of correlation between the pair of diseases (relative risk >1) for over a million pairs where disease 2 (D2) occurs within 5 years of disease 1 (D1) and based on directionality (whether D1->D2 occurs more often than D2->D1, binomial tests).**Disease trajectories combining pairs** with overlapping diagnosis, into three or more diseases.
Lindhagen et al. (2015)	Mortality; change and scale of CCI change	A **state transition model** in discrete time steps to estimate changes in Charlson Comorbidity Index (CCI). Transition probabilities were estimated using **logistic/poisson regression models** for vital status, and CCI changes. **Simulation models** estimated changes in CCI with their confidence intervals.
Lappenschaar et al. (2013)	Cumulative incidence and combinations of six cardiovascular diseases	**Multilevel temporal Bayesian Networks** were used to model the patient's disease status at baseline, and 3-5 years after. The variance induced by the urbanization level, age, and gender, in the multilevel model was explained using Markov Chain Monte Carlo simulation.
Siriwardhana et al. (2018)	Disease state probability and transition probability from a single disease state to a multiple disease state.	**Acyclic multistate model** to define an interconnected progressive chronic disease system for the elderly population. **Aalen and Johansen estimator** (a nonparametric technique) to estimate marginal state occupational probabilities.
Zhu et al. (2018)	Disease progression states and the absorbing state, death.Life-years lost to a specific condition and cumulative lifetime risk of certain conditions	**A disease progression network was constructed** based on the real cohort. One-year progression from state A to B is calculated by counting the number of people who are in state A the previous year and in state B the following year. **Microsimulation** is used to calculate life-years-lost and lifetime risk of particular states.
**Approach 4. Visualisation methods (N=3)**		
Ashworth et al. (2019)	Level of multimorbidity (count of chronic conditions)	**Alluvial plots** based on date of onset of each long-term condition, tabulated as first, second and third to visualise the acquisition sequence
Rocca et al. (2016)	Level of multimorbidity (count of chronic conditions)	The accumulation of multimorbidity was represented graphically using **Aalen-Johansen curves** (a multistate generalization of cumulative incidence curves; unadjusted curves considering all 18 conditions equally).
Xu et al. (2018)	Cumulative incidence of three conditions	**Sankey diagram** was constructed to characterize the dynamic changes of different combinations of the three conditions over time.

[1] IADL = limitations in instrumental activities of daily living. [2] Linear mixed models here refers to any multilevel model for repeated measures over time for each individual, incorporating various labels- mixed linear model, hierarchical models, growth curve model, etc.

For peer review only

The next approach, employed by eight studies[21–23,26,34,36,49,51], focussed on modelling transitions between specific disease states. Six of the studies focused on a limited number of diseases, to make the analysis feasible[22,34,36,37,49,51]. Some studies used principles of state transition modelling, either using Markov principles [51], acyclic multistate models [36] or state transition modelling[23]. Another two studies employed Bayesian techniques including a multilevel temporal Bayesian network [22]and a longest path algorithm to identify the most probable sequence from/to a specific disease following an unsupervised multi-level temporal Bayesian network analysis[34]. One paper derived a disease progression network from real data, and used this for further microsimulation[49]. Finally, two studies used a data-driven approach to create 'temporal disease trajectories' by combining significant temporal directed pairs from all disease pairs possible[21,26]. Transition analysis also enabled the identification of longitudinal clusters[51].

Three studies [20,37,45] used visual methods to describe disease sequences, or multimorbidity acquisition sequences. Ashworth et al [20] used alluvial plots to illustrate multimorbidity acquisition sequences based on date of disease onset, and although useful to understand the order of diseases (co-)occurrence, the visualisations are unable to account for pace of multimorbidity progression. Aalen-Johansen curves (a multistate generalization of cumulative incidence curves) were used to represent the accumulation of multimorbidity graphically [45] and the Sankey diagram to show the longitudinal progression and transitions to each disease and disease combinations[37]. The final approach, employed by seven studies [25,33,40–42,48,54], was to construct meaningful categories of longitudinal multimorbidity patterns (and associate these with other covariates (summarised in Table 4). Methodologies included latent class analysis, latent class growth analysis, growth mixture modelling, or group-based trajectory modelling, and typically identified between four to six groups of distinct longitudinal multimorbidity patterns. Two studies took an associative approach to explore which specific diseases cluster longitudinally [42,48]. For example, Hsu (2015) [48] found four trajectory groups: 'low risk', 'cardiovascular risk only', 'gastrointestinal and chronic non-specific lung disease', and 'multiple risks'. The other five studies focused on stages of accumulation. For example, Hiyoshi et al. (2017) [33]found four trajectory groups ranging from 'a constant low trajectory' to 'a high start and a slow increase trajectory'. Generally, these clusters incorporate data on the initial level of multimorbidity, and accumulation pattern over time, and nearly all showed increased accumulation (the exception being Kim et al [54]which identified some groups with decreasing morbidities).

For peer review only - http://bmjopen.bmj.com/site/about/guidelines.xhtml

Table 4. Methods of studies investigating multimorbidity trajectory groups

Study, year	Statistical approach used	Trajectory groups identified
Hanson et al. (2015)	**Finite mixture modelling** (Proc TRAJ in SAS) was used, with a zero-inflated distribution. Optimal number of groups determined by Bayesian Information Criterion (BIC).	Six groups: 'robust' (no conditions), 'initiates' (none at baseline , increase over time), 'slow initiates' (some at baseline and gradual increase over time), 'accelerated initiates' (none at baseline and quick increase followed by deceleration), 'chronic low' (steady comorbidity over time), 'ailing' (moderate levels of comorbidity at baseline and steady increase over time), 'frail' (high comorbidity at baseline, remaining high over time).
Hiyoshi et al. (2017)	**Group-based trajectory modelling**, using a zero-inflated distribution. Optimal number of groups determined using BIC.	Four groups identified: 'a constant low trajectory', 'a low start and an acute increase trajectory', 'medium start and a slow increase trajectory', 'a high start and a slow increase trajectory'.
Hsu (2015)	**Multiple group-based trajectory model** (Proc TRAJ in SAS) Morbidity was set to follow a logistic model. The optimal group number was determined using the BIC and parsimony principle.	Four chronic disease trajectories were identified: 'low risk', 'cardiovascular risk only', 'gastrointestinal and chronic non-specific lung disease', and 'multiple risks'
Jackson et al. (2015)	**Latent class growth analysis (LCGA)** in Mplus; optimal group number determined using BIC.	Five groups identified: 'no morbidity, constant', 'low morbidity, constant', 'moderate morbidity, constant', 'no morbidity, increasing', and 'low morbidity, increasing'.
Kim et al. (2018)	**Growth mixture modelling (GMM)** in SAS was used; optimal group number determined using BIC.	Five groups identified: 'consistently low', 'increased', 'decreased (low)', 'decreased (high)', and 'consistently high'.
Pugh et al. (2016)	**Latent Class Analysis (LCA)**, based on the distribution of repeated measures in the 20 binary diagnosis outcomes.	Five groups identified for both men and women: 'Healthy', 'Chronic Disease', 'Mental Health', 'Pain' and 'Polytrauma Clinical Triad (PCT: pain, mental health and traumatic brain injury'. Two additional classes found in men were 'Minor Chronic' and 'PCT with chronic disease'.
Strauss et al. (2014)	**LCGA** in Mplus, Optimal group number determined by iterative modelling and BIC values and likelihood ratio test. Morbidity counts were assumed to be Poisson distributed. Quadratic growth curves were applied for all groups identified within the LCGA models.	Five grups identified and validated: no recorded chronic problems', developed a first chronic morbidity over 3 years', 'a developing multimorbidity group', increasing number of chronic morbidities', and 'a multi-chronic group with many chronic morbidities'.

For peer review only - http://bmjopen.bmj.com/site/about/guidelines.xhtml

Results of the studies: outcomes and risk factors

Prediction of other health outcomes

Seven of the studies used multimorbidity trajectories to predict subsequent health outcomes [27,31,38,39,47,49,54], including self-reported health, cognitive ability, disability, medical utilisation, and mortality (Table 5). Among older adults, results showed that an increase in multimorbidity over 10 years was associated with worse reported health [38], and that those who developed multimorbidity faster had greater risk of disability[27]. In one study, changes in multimorbidity were found to be more predictive of mortality than baseline multimorbidity[31]. By contrast, another study confirmed that a change in CCI predicts mortality but not necessarily better than a cross-sectional estimate of multimorbidity[38]. Zhu et al. (2018) [49]found that earlier development of chronic conditions and earlier complications incur greater life-years lost. Finally, multimorbidity accumulation (as a marker of physical health deterioration) predicted faster decline in verbal fluency in older adults without cognitive impairment or dementia[39].

For peer review only - http://bmjopen.bmj.com/site/about/guidelines.xhtml

Table 5. Summary of association analysis for health outcomes related to longitudinal multimorbidity trajectories

Study, year	Outcome investigated	Findings of association analysis
Calderon-Larranaga et al. (2018)	Disability	The speed of multimorbidity is a strong predictor for disability in older adults, even when accounting for baseline number of chronic conditions
Chang et al. (2011)	Medical utilisation	Morbidity strata predicted medical utilization as usefully as more complex risk adjusters.
Fraccaro et al. (2016)	Mortality	Change over time of Charlson Comorbidity Index (CCI) was a stronger predictor of mortality than baseline CCI
Fabbri et al. (2016)	Standardized neurocognitive tests	Accumulation of multimorbidity was associated with faster decline in verbal fluency but seems to have no effect on memory decline, in older adults without mild cognitive impairment or dementia.
Kim et al. (2018)	Mortality	The 'consistently high' multimorbidity trajectory group had the highest risk of mortality at 1, 3 and 5 year follow-ups.
Zeng et al. (2014)	Self-reported health, number of primary care visits, inpatient and emergency admissions, mortality	Growth curve models gave marginally better fitting models for the outcomes of SRH, but mortality and inpatient status was best predicted by multimorbidity snapshot prevalence the year before the survey.
Zhu et al. (2018)	Life expectancy	Diabetes, plus hypertension plus complications reduced life expectancy the most. The earlier the onset of multimorbidity, the greater the reduction in life.

For peer review only

For peer review only - http://bmjopen.bmj.com/site/about/guidelines.xhtml

Risk factors for multimorbidity

Eighteen of selected articles [20,22,24,25,28–30,33,35–37,40,41,43,44,48,50,53] investigated risk factors of multimorbidity trajectories (Table 6). Increasing age, although often accounted for in analyses, emerged as a dominant risk factor for acquisition, worsening or progression of multimorbidity[37,53]. As expected, younger age groups were more likely to belong to a non-chronic healthier cluster[25]. However, trajectories starting with depression were more prevanlent in younger individuals[20]. Younger cohorts were also found to be more likely to develop multimorbidity and to do so at a younger age [35]. A few studies reported gender differences, with conflicting results. While one study found that those in the 'multiple risks' group were more likely to be female [48], another found that men were more likely to transition between disease states than women [36].

Four studies investigated ethnic variations [20,36,43,44]. In two US studies, compared to non-Hispanic whites, Black Americans had a higher rate of multimorbidity at baseline along with a slower rate of disease accumulation over time while Hispanic participants tended to start with fewer diseases and increase more rapidly[43,44]. Different ethnicities also had different disease transition patterns. In the US, white individuals were more likely to transition from ischaemic heart disease to death, while Asian and Native Hawaiian and Pacific Islander individuals were more likely to transition from diabetes to diabetes plus chronic kidney disease[36]. In the UK, disease specific sequences /pathways also differed by ethnicity: For example, the white ethnic group was dominated by depression as a starting point, while diabetes was the most common starting point in the black ethnic group [20].

The studies also explored a range of socio-demographic determinants including area-based deprivation, education, occupation, income, and marital status. Results largely confirm those found with cross-sectional analyses, with lower socioeconomic status associated with worse multimorbidity trajectories. For example, lower levels of education were associated with higher rate of multimorbidity accumulation [29,37] or worse multimorbidity trajectories [41]. People living in more deprived areas were more likely to be in an evolving or multi-chronic multimorbidity cluster [25] and to have trajectories with diabetes and depression as the most common starting point[20].

For peer review only - http://bmjopen.bmj.com/site/about/guidelines.xhtml

Table 6. Summary of association analysis for risk factors related to multimorbidity trajectories

Study, year	Risk factors	Findings of association analysis
Ashworth et al. (2019)	Age, ethnicity, deprivation	Trajectories varied by age, ethnicity, and deprivation. Depression as a starting point was more common in younger, more deprived, and white ethnic group.
Calderon-Larranaga et al. (2019)	Attitude towards life and health	Better attitudes towards life and health were associated with slower multimorbidity development, independent of demographic, clinical, social, personality, and lifestyle factors.
Canizares et al. (2017)	Birth cohort	In each succeeding cohort, multimorbidity rates was higher and multimorbidity emerged earlier. Differences persisted independently of the risk factors for multimorbidity and period effect.
Dekhtyar et al. (2019)	Elementary education (early adulthood), lifelong active occupation (mid-adulthood), social network (later life)	Adults over 60 years old with higher than elementary education, lifelong active occupations and richer social networks had slower multimorbidity accumulation. The association between childhood circumstances and multimorbidity accumulation was attenuated by subsequent (mid- and late-) life experiences. Rich social networks reduced the speed of disease accumulation irrespective of lifelong job stress and level of education.
Fabbri et al. (2015)	Biomarkers: IL-6, IL-1ra, TNF-α receptor II, and DHEAS (as a marker of chronic inflammation and system dysregulation)	Multimorbidity development with age was not linear, and significantly accelerated at older ages. Higher IL-6, IL-1ra, and TNF-α receptor II and low DHEAS were associated with higher multimorbidity at baseline, independent of age, sex, BMI, and education. Higher IL-6 and steeper increase in IL-6 predicted an accelerated rise in multimorbidity over 9 years of follow-up.
Hanson et al. (2015)	Parity, timing of childbearing, birth outcomes of offspring	High parity, early childbearing, and adverse offspring birth outcomes are associated with particular later-life comorbidity patterns and trajectories, when controlling for early-life conditions (age at parental death, childhood socioeconomic status, familial excess longevity, religious participation)
Hiyoshi et al. (2017)	Income, marital status	Income, and physical, cognitive, and psychological function were associated with trajectory group membership in unadjusted analysis but not in fully adjusted analysis.
Hsu (2015)	Gender, education, physical function, depressive symptoms, life satisfaction, number of health examination, smoking, and drinking	Those in the 'multiple risks' group were more likely to be female, less educated, with more physical function difficulties, more depressive symptoms, lower life satisfaction, more health examinations, and not to smoke or drink. Members in the 'CVD risk only' and 'multiple risks' groups were more likely to have physical function difficulties and depressive symptoms.
Jackson et al. (2015)	Overweight or obesity, education, difficulty managing income, smoking alcohol consumption, physical activity	Being overweight or obese, having a lower education level and difficulty managing on income associated with belonging to an accumulation trajectory. Smoking, alcohol intake and physical activity level also appeared to be important risk factors for the development of some trajectories.
Lappenschaar et al. (2013)	Urbanization, multimorbidity at baseline	Urbanization level of a general practice is associated with the higher cumulative incidence of chronic cardiovascular conditions, in particular obesity, hypertension, dyslipidaemia, diabetes mellitus, and ischemic heart disease. Disease accumulation rate higher when multimorbidity is already present at baseline.
Perez et al. (2020)	tGSH (biomarker of multisystem failure)	Lower baseline levels of tGSH were associated with a higher rate of multimorbidity development, independent of covariates.

For peer review only - http://bmjopen.bmj.com/site/about/guidelines.xhtml

Quinones et al. (2011)	Race/ethnicity	White Americans differ from Black and Mexican Americans in terms of level and rate of change of multimorbidity. Mexican Americans demonstrate lower initial levels and slower accumulation of comorbidities relative to White American. In contrast, Black Americans showed an elevated level of multimorbidity throughout the 11-year period of observation, although their rate of change slowed relative to White Americans.
Quinones et al. (2019)	Race/ethnicity	Non-Hispanic black respondents had higher initial chronic disease counts, but slower accumulation rates, than non-Hispanic white respondents. Hispanic respondents had lower initial chronic disease counts but faster accumulation than non-Hispanic white respondents.
Ruel et al. (2014)	Dietary patterns	Greater amount of fruits and vegetables and grain (other than rice and wheat) associated with reduced accumulation of multimorbidity.
Ryan et al. (2018)	Multimorbidity at baseline, age, obesity, gait speed, and grip strength, access to government funded primary care	In non-multimorbid participants age, obesity, gait speed, and grip strength were significantly associated with development of multimorbidity. Age, access to government funded primary care, gait speed, and grip strength were significantly associated with worsening of multimorbidity in those with multimorbidity. Gait speed and age were significantly associated with new condition development in people with complex multimorbidity.<br>-> (Overall) Gait speed, grip strength, and age were significantly associated with both the development of multimorbidity and accrual of additional conditions with evidence of a dose-response relationship.
Siriwardhana et al. (2018)	Age, sex, race/ethnicity	Men were more likely to transition between states than women. Whites had the highest risk of transitioning from ischemic heart disease to death. Asians and Native Hawaiian and Pacific Islanders were more likely to transition from diabetes to diabetes and chronic kidney disease.
Strauss et al. (2014)	Age, deprivation	Younger age groups were more likely to be in the non-chronic cluster than older groups. Females were more likely to develop or start with multimorbidity than males. More deprived individuals were more likely to be in the evolving (rather than static) multimorbidity cluster.
Xu et al. (2018)	Sex, age, marital status, income, education, obesity, physical activity, smoking, and immigrant status.	Odds of multimorbidity progression increased over time and with age. Women with stroke were more likely to progress to another disease and become multimorbid than other baseline characteristics. In adjusted models, accumulation of MM was associated with non-married status, low income, lower education, obesity, sedentary and smoking, immigrant status. Obesity differently associated with different sequences.

For peer review only - http://bmjopen.bmj.com/site/about/guidelines.xhtml

Health and health behaviours also showed associations. A Chinese study showed that a greater consumption of fruits, vegetables and grain slowed the development of multimorbidity[50]. Alcohol consumption, smoking, and physical inactivity was associated with worse multimorbidity trajectory patterns[37,41]. Physical function (measured by gait speed and grip strength at baseline) was associated with development and worsening of multimorbidity over 2 years in a sample of adults aged 50 and over[53]. Being overweight or obese was also associated with developing or worsening multimorbidity trajectory[37,41,53]. Two studies investigated the role of specific biomarkers, finding that chronic inflammation, system dysregulation, and multisystem failure, are associated with faster rate of multimorbidity accumulation[24,30]. There were associations with family factors: being married was found to be protective of greater multimorbidity accumulation[33,48], and young parenthood (younger than 25) and extremely high parity (nine of more births) significant risk factors[40]. Finally, a negative attitude towards life and health such as low life satisfaction and negative health outlook was associated with poorer multimorbidity trajectories[28].

DISCUSSION

Understanding longitudinal multimorbidity trajectories is an important public health priority for clinicians, academics, and funders alike [14]. This review aimed to take a systematic approach to scope existing research in the field with a focus on summarizing commonly used methodological approaches and substantive findings. In doing so we provide, to our knowledge, the first review to address longitudinal studies of multimorbidity, in a field saturated by cross-sectional research [2,10,13,55]. A strength of this review is the systematic and robust approach taken to searching and screening articles for inclusion, and reviewing the selected studies, which should limit selection and extraction bias. We used pre-defined search terms, inclusion criteria, and data extraction tools, and we engaged in double screening and extraction[19]. The scoping review process meant that we summarized a wide variety of evidence, and therefore it was challenging to perform meta-analysis, or use a standardized critical appraisal tool. Nevertheless, we provide a narrative-style critical summary of the selected articles. The results demonstrate that despite widespread expressed interest, relatively few studies do take a longitudinal approach to multimorbidity. All the studies included were published within the last decade and the vast majority using data from high-income countries. The studies showed a great variability in sampling strategy, ways of measuring multimorbidity, and statistical approaches to characterizing multimorbidity longitudinally. Methods for identifying longitudinal patterns ranged from counts of diseases to cluster or group -based analyses, to modeling transitions between diseases or disease sequences, and these were differentially useful for modelling accumulation, sequencing, clusters or transitions. From a

For peer review only - http://bmjopen.bmj.com/site/about/guidelines.xhtml

substantive perspective, the studies showed associations with adverse outcomes such as worse reported health, greater risk of disability, and mortality that we might expect based on the existing cross-sectional research. A range of multimorbidity trajectory risk factors were also identified, including socio-demographic factors, health behaviours, physical function, biomarkers, marriage and fertility factors, and attitudinal factors.

The review has highlighted some geographical bias in the distribution of multimorbidity research. In particular there was an underrepresentation of longitudinal multimorbidity research in low- and middle-income countries (LMICs), which likely reflects the geographical focus of cross-sectional multimorbidity research generally [56]. This may be due to underinvestment in multimorbidity research in LMICs, coupled with challenges of collecting or accessing relevant data. For example, most of selected studies use of electronic medical records or large-scale longitudinal surveys, which are rare in developing countries. Nevertheless, due to the population aging trends in LMICs, multimorbidity is already a major public health issue, with potentially more complex comorbidity patterns (e.g. undiagnosed conditions, or interactions with infectious disease), which deserve research using a longitudinal approach. Recently published work in LMICs countries has tended to employ a cross-sectional design to analyse multimorbidity [57–60], and therefore were not eligible for inclusion in this review. In addition, none of the studies in the review made cross-country comparisons, which may help to generate stronger evidence about disease trajectories and mechanisms involved in multimorbidity development and progression. For example, comparable cross-country patterns may suggest common biological mechanisms, whereas divergent findings could suggest moderation or prevention of disease processes by policy approaches to treatment, health care settings, and institutional structures.

The selected studies used a great variety of data sources including administrative data (primary and secondary care data, health insurance claim data, patient and disease registries) and survey data, leading to variations in sample size and issues of generalisability. Issues of small sample size were only discussed in a limited number of studies, mostly in relation to subgroups such as ethnic minorities [20,33,43,44]. Despite the use of large surveys or administrative data, the majority of studies expressed doubts about the generalisability of their findings. For example, well-educated and wealthy individuals were reported as over-represented in longitudinal survey samples [24,27–29,37,41,53]. Studies using administrative data sources typically investigated multimorbidity based on complete follow-up and excluding those who died, generating immortal time bias and investigating potentially healthier populations [34,47]. In other studies, the choice of data sources

For peer review only - http://bmjopen.bmj.com/site/about/guidelines.xhtml
For peer review only

themselves induced bias, for example where samples were based on health service users[31,42]. Others explained their sample might be representative but only of a particular group in a specific region (e.g. Utah[40]). Another issue of generalisability, mentioned in previous reviews [13], was related to the heterogenous multimorbidity measures used[61]. A wide variety of different diseases were included and only a few studies used 'standard' measurement of multimorbidity like the Charlson [17]or Elixhauser [18] indices. Due to the diversity of data sources, diseases were ascertained in multiple ways, using clinical-diagnosis, laboratory results, medication use, and self-report. The only common measurement feature was that studies in this review tended not to define multimorbidity as the presence or two or more diseases.

The choice of statistical methods served to highlight or obscure different aspects of multimorbidity. For example, the most common approach, multi- or single-level regression modelling, emphasizes accumulation, providing the opportunity to simultaneously evaluate the baseline level of multimorbidity and the (slope) change in multimorbidity, and how this differs between groups with different characteristics. However, it tends to obscure the role of specific diseases by collapsing all morbidity in a single count or index, and we cannot tell, for example, whether this faster accumulation is predominantly occurring among certain types of disorders. Complementary to regression approaches, grouped-based methodologies aimed to classify individuals into types of multimorbidity accumulation. A minority of studies employed the cluster-based approach to understand how specific diseases co-occur over time [42,48], which extends cross-sectional approaches often referred to as associative multimorbidity [9]. This has the advantage of providing a more detailed understanding of the constellation of diseases that contribute to distinct trajectories, but due to the rarity of some diseases, will tend to find distinguish only highly prevalent clusters, and is not suitable for rarer more disease trajectories.

Some studies conceptualised longitudinal multimorbidity as transitioning between different disease states, utilizing either structured Markov frameworks [51], multistate modelling [23,36] or a more data-driven, unsupervised approaches[21,24]. The former, more structured approach to disease transition tended to provide a very detailed understanding of interactions between a small set of diseases, which can provide useful evidence for targeting prevention at those with the first disease, a risk stratification approach. The latter, data-driven approaches provide very comprehensive evidence for population-based strategies, but relies on large datasets collected over a number of years, and appropriate clinical expertise to interpret the results of patterns identified through artificial intelligence (AI). Given the growing interdisciplinary collaborations between epidemiology

For peer review only - http://bmjopen.bmj.com/site/about/guidelines.xhtml

and computer science, data-driven research will continue to expand in the coming years and extend to prediction modelling and projections. One of the strengths of computer science, and the recent new developments in AI with machine learning, is the ability to work towards solutions that can combine prediction models and compare different treatment options for cohorts of patients (e.g. what is the likelihood that a medication commonly used for one chronic condition may speed up the progression of another condition or lead to the development of a new condition).

Compared with cross-sectional studies, longitudinal approaches provide more detailed insight about the role of specific risk factors. For example, while age is a known risk factor, this review highlights how older individuals, once multimorbid, show acceleration of multimorbidity [53]. Multimorbidity trajectory patterns varied by ethnicity [20,36,43,44], marital status [33,37], educational level and area-level deprivation [25,29,37,41], confirming some patterns observed in cross-sectional data. A useful exploitation of longitudinal data – not included in these studies- would be to explore how change in risk factors such as SES or marital status influences different multimorbidity trajectories, which may help identify at-risk groups and target prevention strategies. As highlighted by Zhu and colleagues [49], the earlier the multimorbidity onset in the life course, the greater the life year lost for that individual. Therefore, future research should seek to take a life course approach in order to disentangle early preventable factors of multimorbidity onset but also to determine later life factors influencing additional disease accumulation. Risk factors should be considered at the level of the individual (life course and contemporaneous factors), medication use, and the wider social environment, including poor environmental conditions, and interaction with institutional structures (e.g. health care system organization). The increasing availability of 'big data' which links longitudinal administrative data on individuals with health and geospatial data will make these holistic approaches technically possible. Future research should focus on generating the knowledge required to develop interventions aimed at preventing both the onset and the worsening of multimorbidity.

For peer review only

CONCLUSION

This review identifies a small but developing body of literature attempting to describe multimorbidity longitudinally. There was a notable lack of studies in low and middle-income countries, as well exploring minority ethnic groups. A wide variety of complementary methods are employed, which emphasizing factors associated with greater disease accumulation, speed of accumulation, and specific disease transition processes. Methodologies based on disease ordering or sequence was seldom explored by the studies, and while it is challenging to identify exact timing of disease, future research could seek to investigate disease sequencing that underlies the

For peer review only - http://bmjopen.bmj.com/site/about/guidelines.xhtml

accumulation process. Risk factors for trajectory types could inform future intervention and prevention strategies at critical life course periods and disease progression turning points. Initiatives to enable researchers greater access to relevant data sources, such as the HDR UK initiative to harmonise datasets for multimorbidity research, is crucial and should become more generalised in order to gain the insight on multimorbidity processes required to feed into prevention and policy makers strategies at a global scale.

Fig 1 Caption:

Figure 1. Study selection process

AUTHOR CONTRIBUTIONS

GC participated in the screening, data extraction, and analysis and helped draft the manuscript. CM participated in the screening, data extraction, and analysis and helped draft the manuscript. FS and helped draft the manuscript. KK acquired the funding, conceptualised the idea for the study, participated in the screening, data extraction, and analysis and finalised the manuscript. All authors approved the final version for submission.

ACKNOWLEDGEMENTS

We thank Iris Ho and Bruce Guthrie for providing additional references that our systematic search might have missed.

FUNDING

This work was supported by the Academy of Medical Sciences (AMS), the Wellcome Trust, the Government Department of Business, Energy and Industrial Strategy (BEIS), the British Heart Foundation Diabetes UK, and the Global Challenges Research Fund (GCRF) [Grant number SBF004\1093 awarded to Katherine Keenan]. The funders had no input to the design or execution of the study.

DECLARATIONS OF INTEREST

None.

For peer review only

REFERENCES

1 Diederichs C, Berger K, Bartels DB. The Measurement of Multiple Chronic Diseases--A Systematic Review on Existing Multimorbidity Indices. *Journals Gerontol Ser A Biol Sci Med Sci* 2011;**66A**:301–11. doi:10.1093/gerona/glq208

2 Johnston MC, Crilly M, Black C, *et al.* Defining and measuring multimorbidity: a systematic review of systematic reviews. *Eur J Public Health* 2019;**29**:182–9. doi:10.1093/eurpub/cky098

3 Kingston A, Robinson L, Booth H, *et al.* Projections of multi-morbidity in the older population in England to 2035: estimates from the Population Ageing and Care Simulation (PACSim) model. *Age Ageing* 2018;**47**:374–80. doi:10.1093/ageing/afx201

4 Makovski TT, Schmitz S, Zeegers MP, *et al.* Multimorbidity and quality of life: Systematic literature review and meta-analysis. Ageing Res. Rev. 2019;**53**:100903. doi:10.1016/j.arr.2019.04.005

5 Ryan A, Wallace E, O'Hara P, *et al.* Multimorbidity and functional decline in community-dwelling adults: A systematic review. *Health Qual Life Outcomes* 2015;**13**:1–13. doi:10.1186/s12955-015-0355-9

6 Nunes BP, Flores TR, Mielke GI, *et al.* Multimorbidity and mortality in older adults: A systematic review and meta-analysis. Arch. Gerontol. Geriatr. 2016;**67**:130–8. doi:10.1016/j.archger.2016.07.008

7 Wang L, Si L, Cocker F, *et al.* A Systematic Review of Cost-of-Illness Studies of Multimorbidity. Appl. Health Econ. Health Policy. 2018;**16**:15–29. doi:10.1007/s40258-017-0346-6

8 Ng SK, Tawiah R, Sawyer M, *et al.* Patterns of multimorbid health conditions: a systematic review of analytical methods and comparison analysis. *Int J Epidemiol* 2018;**47**:1687–704. doi:10.1093/ije/dyy134

9 Prados-Torres A, Calderón-Larrañaga A, Hancco-Saavedra J, *et al.* Multimorbidity patterns: a systematic review. *J Clin Epidemiol* 2014;**67**:254–66. doi:10.1016/J.JCLINEPI.2013.09.021

10 Violan C, Foguet-Boreu Q, Flores-Mateo G, *et al.* Prevalence, Determinants and Patterns of Multimorbidity in Primary Care: A Systematic Review of Observational Studies. *PLoS One* 2014;**9**:e102149. doi:10.1371/journal.pone.0102149

11 Pathirana TI, Jackson CA. Socioeconomic status and multimorbidity: a systematic review and meta-analysis. *Aust N Z J Public Health* 2018;**42**:186–94. doi:10.1111/1753-6405.12762

12 Fortin M, Haggerty J, Almirall J, *et al.* Lifestyle factors and multimorbidity: A cross sectional study. *BMC Public Health* 2014;**14**:1–8. doi:10.1186/1471-2458-14-686

13 Xu X, Mishra GD, Jones M. Mapping the global research landscape and knowledge gaps on multimorbidity: a bibliometric study. *J Glob Health* 2017;**7**:010414.

For peer review only - http://bmjopen.bmj.com/site/about/guidelines.xhtml

doi:10.7189/jogh.07.010414

14 Academy of Medical Sciences. Multimorbidity: A Priority for Global Health Research. London: 2018. https://acmedsci.ac.uk/file-download/82222577

15 Munn Z, Peters MDJ, Stern C, *et al.* Systematic review or scoping review? Guidance for authors when choosing between a systematic or scoping review approach. *BMC Med Res Methodol* 2018;**18**:1–7. doi:10.1186/s12874-018-0611-x

16 Tricco AC, Lillie E, Zarin W, *et al.* PRISMA Extension for Scoping Reviews (PRISMA-ScR): Checklist and Explanation. *Ann Intern Med* 2018;**169**:467. doi:10.7326/M18-0850

17 Charlson ME, Pompei P, Ales KL, *et al.* A new method of classifying prognostic comorbidity in longitudinal studies: Development and validation. *J Chronic Dis* 1987;**40**:373–83. doi:10.1016/0021-9681(87)90171-8

18 Elixhauser A, Steiner C, Harris DR, *et al.* Comorbidity Measures for Use with Administrative Data. *Med Care* 1998;**36**:8–27. doi:10.1097/00005650-199801000-00004

19 Buscemi N, Hartling L, Vandermeer B, *et al.* Single data extraction generated more errors than double data extraction in systematic reviews. *J Clin Epidemiol* 2006;**59**:697–703. doi:10.1016/j.jclinepi.2005.11.010

20 Ashworth M, Durbaba S, Whitney D, *et al.* Journey to multimorbidity: Longitudinal analysis exploring cardiovascular risk factors and sociodemographic determinants in an urban setting. *BMJ Open* 2019;**9**:e031649. doi:10.1136/bmjopen-2019-031649

21 Jensen AB, Moseley PL, Oprea TI, *et al.* Temporal disease trajectories condensed from population-wide registry data covering 6.2 million patients. *Nat Commun* 2014;**5**:4022. doi:10.1038/ncomms5022

22 Lappenschaar M, Hommersom A, Lucas PJF, *et al.* Multilevel temporal Bayesian networks can model longitudinal change in multimorbidity. *J Clin Epidemiol* 2013;**66**:1405–16. doi:10.1016/j.jclinepi.2013.06.018

23 Lindhagen L, Van Hemelrijck M, Robinson D, *et al.* How to model temporal changes in comorbidity for cancer patients using prospective cohort data. *BMC Med Inform Decis Mak* 2015;**15**:96. doi:10.1186/s12911-015-0217-8

24 Pérez LM, Hooshmand B, Mangialasche F, *et al.* Glutathione Serum Levels and Rate of Multimorbidity Development in Older Adults. *Journals Gerontol Ser A* 2020;**75**:1089–94. doi:10.1093/gerona/glz101

25 Strauss VY, Jones PW, Kadam UT, *et al.* Distinct trajectories of multimorbidity in primary care were identified using latent class growth analysis. *J Clin Epidemiol* 2014;**67**:1163–71. doi:10.1016/j.jclinepi.2014.06.003

For peer review only - http://bmjopen.bmj.com/site/about/guidelines.xhtml

26 Beck MK, Jensen AB, Nielsen AB, *et al.* Diagnosis trajectories of prior multi-morbidity predict sepsis mortality. *Sci Rep* 2016;**6**:1–9. doi:10.1038/srep36624

27 Calderón-Larrañaga A, Santoni G, Wang HX, *et al.* Rapidly developing multimorbidity and disability in older adults: does social background matter? *J Intern Med* 2018;**283**:489–99. doi:10.1111/joim.12739

28 Calderón-Larrañaga A, Vetrano DL, Welmer AK, *et al.* Psychological correlates of multimorbidity and disability accumulation in older adults. *Age Ageing* 2019;**48**:789–96. doi:10.1093/ageing/afz117

29 Dekhtyar S, Vetrano DL, Marengoni A, *et al.* Association Between Speed of Multimorbidity Accumulation in Old Age and Life Experiences: A Cohort Study. *Am J Epidemiol* 2019;**188**:1627–36. doi:10.1093/aje/kwz101

30 Fabbri E, An Y, Zoli M, *et al.* Aging and the Burden of Multimorbidity: Associations With Inflammatory and Anabolic Hormonal Biomarkers. *Journals Gerontol Ser A* 2015;**70**:63–70. doi:10.1093/gerona/glu127

31 Fraccaro P, Kontopantelis E, Sperrin M, *et al.* Predicting mortality from change-over-time in the Charlson Comorbidity Index: A retrospective cohort study in a data-intensive UK health system. *Med (United States)* 2016;**95**. doi:10.1097/MD.0000000000004973

32 Gellert P, Von Berenberg P, Oedekoven M, *et al.* Centenarians Differ in Their Comorbidity Trends during the 6 Years before Death Compared to Individuals Who Died in Their 80s or 90s. *Journals Gerontol - Ser A Biol Sci Med Sci* 2018;**73**:1357–62. doi:10.1093/gerona/glx136

33 Hiyoshi A, Fall K, Bergh C, *et al.* Comorbidity trajectories in working age cancer survivors: A national study of Swedish men. *Cancer Epidemiol* 2017;**48**:48–55. doi:10.1016/j.canep.2017.03.001

34 Faruqui SHA, Alaeddini A, Jaramillo CA, *et al.* Mining patterns of comorbidity evolution in patients with multiple chronic conditions using unsupervised multi-level temporal Bayesian network. *PLoS One* 2018;**13**:e0199768. doi:10.1371/journal.pone.0199768

35 Canizares M, Hogg-Johnson S, Gignac MAM, *et al.* Increasing trajectories of multimorbidity over time: Birth cohort differences and the role of changes in obesity and income. *Journals Gerontol - Ser B Psychol Sci Soc Sci* 2018;**73**:1303–14. doi:10.1093/geronb/gbx004

36 Siriwardhana C, Lim E, Davis J, *et al.* Progression of diabetes, ischemic heart disease, and chronic kidney disease in a three chronic conditions multistate model. *BMC Public Health* 2018;**18**:752. doi:10.1186/s12889-018-5688-y

37 Xu X, Mishra GD, Dobson AJ, *et al.* Progression of diabetes, heart disease, and stroke multimorbidity in middle-aged women: A 20-year cohort study. *PLoS Med* 2018;**15**:e1002516.

For peer review only

doi:10.1371/journal.pmed.1002516

38 Zeng C, Ellis JL, Steiner JF, *et al.* Assessment of morbidity over time in predicting health outcomes. *Med Care* 2014;**52**. doi:10.1097/MLR.0000000000000033

39 Fabbri E, An Y, Zoli M, *et al.* Association Between Accelerated Multimorbidity and Age-Related Cognitive Decline in Older Baltimore Longitudinal Study of Aging Participants without Dementia. *J Am Geriatr Soc* 2016;**64**:965–72. doi:10.1111/jgs.14092

40 Hanson HA, Smith KR, Zimmer Z. Reproductive History and Later-Life Comorbidity Trajectories: A Medicare-Linked Cohort Study From the Utah Population Database. *Demography* 2015;**52**:2021–49. doi:10.1007/s13524-015-0439-5

41 Jackson CA, Dobson A, Tooth L, *et al.* Body mass index and socioeconomic position are associated with 9-year trajectories of multimorbidity: A population-based study. *Prev Med (Baltim)* 2015;**81**:92–8. doi:10.1016/j.ypmed.2015.08.013

42 Pugh MJ, Finley EP, Wang CP, *et al.* A retrospective cohort study of comorbidity trajectories associated with traumatic brain injury in veterans of the Iraq and Afghanistan wars. *Brain Inj* 2016;**30**:1481–90. doi:10.1080/02699052.2016.1219055

43 Quiñones AR, Botoseneanu A, Markwardt S, *et al.* Racial/ethnic differences in multimorbidity development and chronic disease accumulation for middle-aged adults. *PLoS One* 2019;**14**:e0218462. doi:10.1371/journal.pone.0218462

44 Quinones AR, Liang J, Bennett JM, *et al.* How Does the Trajectory of Multimorbidity Vary Across Black, White, and Mexican Americans in Middle and Old Age? *Journals Gerontol Ser B Psychol Sci Soc Sci* 2011;**66B**:739–49. doi:10.1093/geronb/gbr106

45 Rocca WA, Gazzuola-Rocca L, Smith CY, *et al.* Accelerated Accumulation of Multimorbidity After Bilateral Oophorectomy: A Population-Based Cohort Study. *Mayo Clin Proc* 2016;**91**:1577–89. doi:10.1016/j.mayocp.2016.08.002

46 Ruel G, Lévesque J-F, Stocks N, *et al.* Understanding the Evolution of Multimorbidity: Evidences from the North West Adelaide Health Longitudinal Study (NWAHS). *PLoS One* 2014;**9**:e96291. doi:10.1371/journal.pone.0096291

47 Chang HY, Clark JM, Weiner JP. Morbidity trajectories as predictors of utilization: Multi-year disease patterns in taiwan's national health insurance program. *Med Care* 2011;**49**:918–23. doi:10.1097/MLR.0b013e31821b344f

48 Hsu HC. Trajectories of multimorbidity and impacts on successful aging. *Exp Gerontol* 2015;**66**:32–8. doi:10.1016/j.exger.2015.04.005

49 Zhu Z, Heng BH, Teow KL. Lifetime trajectory simulation of chronic disease progression and comorbidity development. *J Biomed Inform* 2018;**88**:29–36. doi:10.1016/j.jbi.2018.11.002

For peer review only - http://bmjopen.bmj.com/site/about/guidelines.xhtml

50 Ruel G, Shi Z, Zhen S, *et al.* Association between nutrition and the evolution of multimorbidity: The importance of fruits and vegetables and whole grain products. *Clin Nutr* 2014;**33**:513–20. doi:10.1016/j.clnu.2013.07.009

51 Alaeddini A, Jaramillo CA, Faruqui SHA, *et al.* Mining major transitions of chronic conditions in patients with multiple chronic conditions. *Methods Inf Med* 2017;**56**:391–400. doi:10.3414/ME16-01-0135

52 Ruel G, Lévesque JF, Stocks N, *et al.* Understanding the evolution of multimorbidity: Evidences from the North West Adelaide Health Longitudinal Study (NWAHS). *PLoS One* 2014;**9**:e96291. doi:10.1371/journal.pone.0096291

53 Ryan A, Murphy C, Boland F, *et al.* What Is the Impact of Physical Activity and Physical Function on the Development of Multimorbidity in Older Adults Over Time? A Population-Based Cohort Study. *Journals Gerontol Ser A* 2018;**73**:1538–44. doi:10.1093/gerona/glx251

54 Kim JH, Rhee Y, Lee Y. Longitudinal changes in comorbidity patterns over time in relation to mortality in older Korean adults. *Arch Gerontol Geriatr* 2018;**79**:63–8. doi:10.1016/j.archger.2018.08.004

55 Nguyen H, Manolova G, Daskalopoulou C, *et al.* Prevalence of multimorbidity in community settings: A systematic review and meta-analysis of observational studies. *J Comorbidity* 2019;**9**:2235042X1987093. doi:10.1177/2235042x19870934

56 Abebe F, Schneider M, Asrat B, *et al.* Multimorbidity of chronic non-communicable diseases in low- and middle-income countries: A scoping review. *J Comorbidity* 2020;**10**:2235042X2096191. doi:10.1177/2235042x20961919

57 Bayes-Marin I, Sanchez-Niubo A, Egea-Cortés L, *et al.* Multimorbidity patterns in low-middle and high income regions: A multiregion latent class analysis using ATHLOS harmonised cohorts. *BMJ Open* 2020;**10**:34441. doi:10.1136/bmjopen-2019-034441

58 Garin N, Koyanagi A, Chatterji S, *et al.* Global Multimorbidity Patterns: A Cross-Sectional, Population-Based, Multi-Country Study. *Journals Gerontol Ser A Biol Sci Med Sci* 2016;**71**:205–14. doi:10.1093/gerona/glv128

59 Pati S, Swain S, Metsemakers J, *et al.* Pattern and severity of multimorbidity among patients attending primary care settings in Odisha, India. *PLoS One* 2017;**12**:e0183966. doi:10.1371/journal.pone.0183966

60 Rivera-Almaraz A, Manrique-Espinoza B, Ávila-Funes JA, *et al.* Disability, quality of life and all-cause mortality in older Mexican adults: Association with multimorbidity and frailty. *BMC Geriatr* 2018;**18**:1–9. doi:10.1186/s12877-018-0928-7

61 Stirland LE, González-Saavedra L, Mullin DS, *et al.* Measuring multimorbidity beyond counting

For peer review only

diseases: Systematic review of community and population studies and guide to index choice. *BMJ* 2020;**368**. doi:10.1136/bmj.m160

For peer review only

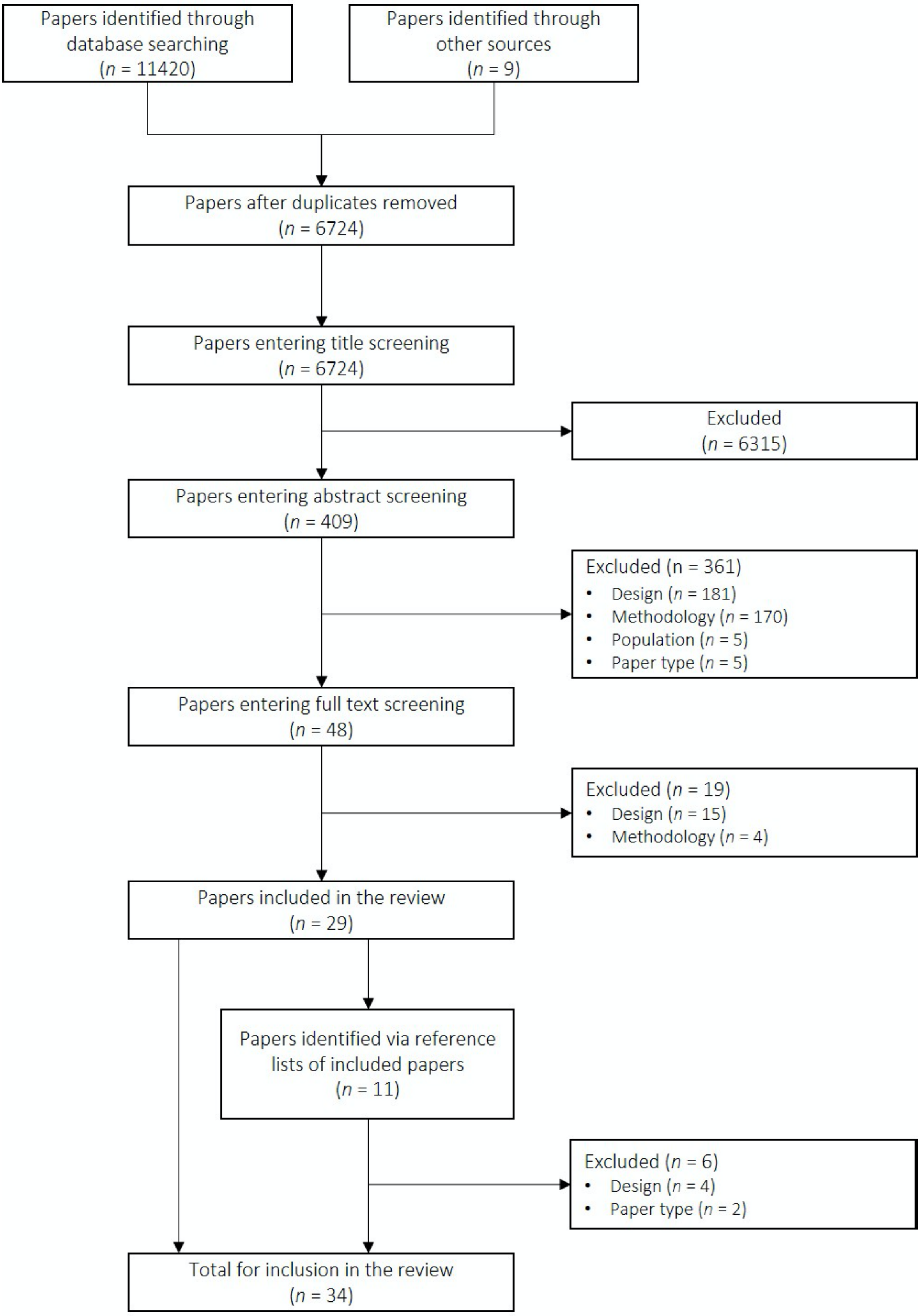
Papers identified through database searching (n = 11420)
Papers identified through other sources (n = 9)
Papers after duplicates removed (n = 6724)
Papers entering title screening (n = 6724)
Excluded (n = 6315)
Papers entering abstract screening (n = 409)
Excluded (n = 361)
• Design (n = 181)
• Methodology (n = 170)
• Population (n = 5)
• Paper type (n = 5)
Papers entering full text screening (n = 48)
Excluded (n = 19)
• Design (n = 15)
• Methodology (n = 4)
Papers included in the review (n = 29)
Papers identified via reference lists of included papers (n = 11)
Excluded (n = 6)
• Design (n = 4)
• Paper type (n = 2)
Total for inclusion in the review (n = 34)

Appendix A: Data Search Strategies

Medline

1. exp comorbidity/
2. exp multiple chronic conditions/
3. (co-morbid* or comorbid* or multi-morbid* or multimorbid or co-occur* or cooccur*).mp.
4. 1 or 2 or 3
5. exp cohort studies/
6. 4 and 5
7. ((disease or condition or illness) and (cluster* or trajector* or cascade* or accumulat* or combination* or sequenc* or transition*)).mp.
8. 6 and 7
9. limit 8 to (english language and humans and ("adult (19 to 44 years)" or "young adult and adult (19-24 and 19-44)" or "middle age (45 to 64 years)" or "middle aged (45 plus years)" or "all aged (65 and over)" or "aged (80 and over)"))
10. 9 not (dna or cell* or gene or genes or bater* or covid*).mp.

Web of Science

1. TOPIC: (co-morbid* or comorbid* or multi-morbid* or multimorbid* or co-occur* or cooccur*)
2. TOPIC: (cohort* or longitudinal* or prospective*)
3. TOPIC: ((disease or condition or illness) and (cluster* or trajector* or cascade* or accumulat* or combination* or sequenc* or transition*))
4. 1 and 2
5. 3 and 4
6. 5 Refined by: Web of Science Index: (WOS.SCI or WOS.SSCI) and Languages: (English) and Document type: (Article)
7. Topic: (cell* or gene or genes or bacter* or DNA or covid*)
8. 6 not 7

Embase

1. (co-morbid* or comorbid* or multi-morbid* or multimorbid or co-occur* or cooccur*).mp.
2. exp multiple chronic conditions/
3. 1 or 2
4. prospective study/
5. cohort study/
6. exp longitudinal study/
7. 4 or 5 or 6
8. 3 and 7
9. ((disease or condition or illness) and (cluster* or trajector* or cascade* or accumulat* or combination* or sequenc* or transition*)).mp.
10. 8 and 9
11. limit 10 to (human and english language and (article or article in press) and (adult <18 to 64 years> or aged <65+ years>))
12. 11 not (dna or cell* or bacter* or gene or genes or covid*).mp.

Scopus

For peer review only

```
( ( TITLE-ABS-KEY ( co-morbid*  OR  comorbid*  OR  multi-
morbid*  OR  multimorbid*  OR  co-occur*  OR  cooccur* ) )  AND  ( TITLE-ABS-
KEY ( ( disease  OR  condition  OR  illness )  AND  ( cluster*  OR  trajector*  OR  cascade
*  OR  accumulat*  OR  combination*  OR  sequenc*  OR  transition* ) ) )  AND  ( TITLE-
ABS-KEY ( cohort*  OR  longitudinal*  OR  prospective* ) ) )  AND NOT  ( TITLE-ABS
KEY ( cell*  OR  gene  OR  genes  OR  bacter*  OR  dna  OR  covid* ) )  AND  ( LIMIT-
TO ( DOCTYPE ,  "ar" ) )  AND  ( LIMIT-TO ( SUBJAREA ,  "MEDI" )  OR  LIMIT-
TO ( SUBJAREA ,  "PSYC" )  OR  LIMIT-TO ( SUBJAREA ,  "NURS" )  OR  LIMIT-
TO ( SUBJAREA ,  "MULT" )  OR  LIMIT-TO ( SUBJAREA ,  "HEAL" )  OR  LIMIT-
TO ( SUBJAREA ,  "SOCI" )  OR  LIMIT-TO ( SUBJAREA ,  "COMP" )  OR  LIMIT-
TO ( SUBJAREA ,  "DECI" ) )  AND  ( LIMIT-
TO ( EXACTKEYWORD ,  "Human" )  OR  LIMIT-
TO ( EXACTKEYWORD ,  "Humans" ) )  AND  ( LIMIT-
TO ( LANGUAGE ,  "English" ) )  AND  ( LIMIT-TO ( SRCTYPE ,  "j" ) )
```

For peer review only

Appendix B: List of conditions included in each selected study

Study, year	List of diseases or disease categories included if available
Alaeddini et al. (2017)	Back pain, hypertension, Post-Traumatic Stress Disorder (PTSD), and depression
Ashworth et al. (2019)	Long-term conditions: atrial fibrillation, COPD, chronic pain, Chronic Kidney Disease (CKD), Coronary Heart Disease (CHD), Diabetes Mellitus (DM), dementia, depression, Heart Failure (HF), serious mental illness, stroke, and morbid obesity
Beck et al. (2016)	All temporal trajectory of four diseases, identified from temporal directed pairs of diseases combined into longer trajectories of temporal consecutives diseases (three temporal disease pairs) with sepsis as the fourth disease, trajectory must be found for at least 20 patients following Jensen (2014) methodology
Calderon-Larranaga et al. (2018)	918 chronic conditions identified by an international team of geriatricians, general practitioners and epidemiologists. A condition was selected if defined as chronic and if it either worsened quality of life, residual disability remained or if long period of care, treatment or rehabilitation was required.
Calderon-Larranaga et al. (2019)	As specified by Calderon-Larranaga et al. (2018)
Canizares et al. (2017)	Chronic conditions: arthritis, back problems, asthma, allergies (excluding food allergies), bronchitis, emphysema, diabetes, high blood pressure, heart conditions, stroke, cancer, ulcers, urinary incontinency, dementia, migraine, glaucoma, and cataracts.
Chang et al. (2011)	27 morbidity patterns grouped into 6 trajectory groups: constant high, constant medium, constant low, decreasing, increasing, and erratic.
Dekhtyar et al. (2019)	As specified by Calderon-Larranaga et al. (2018)
Fabbri et al. (2015)	Chronic conditions: hypertension, diabetes, ischemic heart disease, congestive heart failure, stroke, COPD, cancer, Parkinson's disease, hip fracture, lower extremities joint disease, anemia, chronic kidney disease, peripheral arterial disease, cognitive impairment, and depression.
Fabbri et al. (2016)	Chronic diseases: hypertension, diabetes, ischemic heart disease, congestive heart failure, stroke, COPD, cancer, Parkinson's disease, hip fracture, lower extremities joint disease, anemia, chronic kidney disease, and peripheral arterial disease.
Faruqui et al. (2018)	Traumatic brain injury (TBI), Post-Traumatic Stress Disorder (PTSD), depression, substance abuse, and back pain.
Fraccaro et al. (2016)	A list of 22 diseases of the CCI score is used but due to data restrictions on sexual and mental health, diseases such as HIV and dementia could not be included
Gellert et al. (2018)	A list of 30 health conditions based on the Elixhauser multimorbidity index
Hanson et al. (2015)	A list of 17 conditions based on the Charlson comorbidity index: myocardial infarction, congestive heart failure, peripheral vascular disease, cerebrovascular disease, dementia, chronic pulmonary disease, rheumatologic disease, peptic ulcer disease, mild liver disease, diabetes (mild to moderate), diabetes with chronic complications, hemiplegia or paraplegia, renal kidney disease, any malignancy, moderate or severe liver disease, metastatic solid tumor, AIDS
Hiyoshi et al. (2017)	List of diseases including some based on the Charlson Comorbidity Index: depression, anxiety, osteoporosis, and infectious disease + myocardial infarction, congestive heart failure, peripheral vascular disease, cerebrovascular disease, dementia, chronic pulmonary disease, rheumatologic disease, chronic renal failure, mild liver disease, diabetes with and without chronic complications, hemiplegia or paraplegia, renal disease, moderate or severe liver disease, and AIDS/HIV
Hsu (2015)	Diseases were grouped into six disease types: CVD (including diabetes mellitus, heart disease, and stroke), chronic non-specific lung disease (CNSLD, including lung disease, asthma, bronchitis, and emphysema), gastrointestinal disease (GI, including liver disease, gallbladder disease, and gastrointestinal disorders), arthritis (or rheumatism), cancer, and renal disease.
Jackson et al. (2015)	Diabetes, impaired glucose tolerance, osteoarthritis, rheumatoid arthritis, other arthritis, heart disease, hypertension, stroke, asthma, bronchitis or emphysema, osteoporosis, breast cancer, cervical cancer, other cancer, depression, anxiety or nervous disorder, and other psychiatric condition and chronic fatigue syndrome

For peer review only - http://bmjopen.bmj.com/site/about/guidelines.xhtml

Jensen et al. (2014)	All diseases taken at three level ICD 10 code
Kim et al. (2018)	Metastatic cancer, congestive heart failure, dementia, renal failure, weight loss, hemiplegia, alcohol abuse, any tumour, cardiac arrhythmias, chronic pulmonary disease, coagulopathy, complicated diabetes, deficiency anaemias, fluid and electrolyte disorders, liver disease, peripheral vascular disorder, psychosis, pulmonary circulation disorders, HIV/AIDS, hypertension
Lappenschaar et al. (2013)	Diabetes mellitus, heart failure, stroke, ischemic heart disease, retinopathy, and nephropathy<br>Obesity, hypertension, and lipid disorder used as health risk
Lindhagen et al. (2015)	A list of 17 groups of diseases based on the Charlson Comorbidity Index
Perez et al. (2020)	As specified by Calderon-Larranaga et al. (2018)
Pugh et al. (2016)	Traumatic brain injury and a list of diseases with include "physical thyroid, obesity, hypertension, diabetes, chronic lung disease, cardiac conditions) and mental health (depression, anxiety, SUD, PTSD, bipolar disorder) conditions as well as other conditions such as hearing/tinnitus/vision disorders, headache, other pain, insomnia, memory loss, dementia, seizures, spinal cord injury/amputations/burns.
Quinones et al. (2011)	Hypertension, heart disease, diabetes, cancer, lung disease, arthritis, and stroke
Quinones et al. (2019)	Heart disease (including myocardial infarction, coronary heart disease, angina, congestive heart failure, or other heart problems), hypertension, stroke (excluding transient ischemic attack), diabetes, arthritis, lung disease (including chronic bronchitis or emphysema and excluding asthma), and cancer (including any malignant tumours with the exception of skin cancer).
Rocca et al. (2016)	Depression, anxiety, substance abuse disorders, dementia, schizophrenia, hyperlipidemia, hypertension, diabetes, cardiac arrhythmia, coronary artery disease, stroke, congestive heart failure, arthritis, cancer (all types), asthma, chronic obstructive pulmonary disease, osteoporosis, chronic kidney disease.
Ruel et al. (2014)	Asthma, cardiovascular disease & stroke (CVD), chronic obstructive pulmonary disease (COPD), diabetes, mood and anxiety disorders, any other mental disorders, hypercholesterolemia, and hypertension.
Ruel et al. (2014)	Anemia, hypertension, hypercholesterolemia, diabetes, arthritis, hepatitis, coronary heart disease, asthma, stroke, fracture, and cancer.
Ryan et al. (2018)	The list of 16 chronic conditions groups: cardiac conditions, cerebrovascular conditions, hypertension, diabetes, high cholesterol, chronic respiratory disease, liver disease, eye disease, cognitive impairment, arthritis, osteoporosis, cancer, Parkinson's disease, emotional/psychological condition, stomach ulcers, and varicose veins.
Siriwardhana et al. (2018)	Diabetes, ischemic heart<br>disease, and chronic kidney disease
Strauss et al. (2014)	A list of 42 chronic and progressive morbidities as validated by a clinical consensus exercise with eight GPs: benign neoplasm of prostate, carcinoma in situ, neurofibromatosis, hypothyroidism, diabetes mellitus, pure hypercholesterolaemia, obesity, hereditary haemolytic anaemia, senile/presenile dementia, hereditary and idiopathic peripheral neuropathy, primary angle glaucoma, cataract, corneal opacity and disorders of cornea, deafness, mitral stenosis, rheumatic heart disease, high blood pressure, hypertensive heart disease, hypertensive renal disease, angina pectoris, ischaemic heart disease, atrial fibrillation, congestive heart failure, cerebral atherosclerosis, cerebrovascular disease, intermittent claudication, chronic bronchitis, emphysema, extrinsic allergic alveolitis, pulmonary oedema, diffuse pulmonary fibrosis, nephritis and nephropathy, hydronephrosis, prostatism, rheumatoid arthritis, arthropathy, cervical spondylosis, Paget's disease of bone, osteoporosis, Nervous system injury, war injuries
Xu et al. (2018)	Diabetes mellitus, heart disease (heart attack, angina), and stroke
Zeng et al. (2014)	The list of 17 chronic conditions of the Charlson Comorbidity Index
Zhu et al. (2018)	Diabetes type 2, hypertension, chronic kidney disease, coronary heart disease, stroke, and heart failure

For peer review only - http://bmjopen.bmj.com/site/about/guidelines.xhtml

Preferred Reporting Items for Systematic reviews and Meta-Analyses extension for Scoping Reviews (PRISMA-ScR) Checklist

SECTION	ITEM	PRISMA-ScR CHECKLIST ITEM	REPORTED ON PAGE #
**TITLE**			
Title	1	Identify the report as a scoping review.	1
**ABSTRACT**			
Structured summary	2	Provide a structured summary that includes (as applicable): background, objectives, eligibility criteria, sources of evidence, charting methods, results, and conclusions that relate to the review questions and objectives.	2
**INTRODUCTION**			
Rationale	3	Describe the rationale for the review in the context of what is already known. Explain why the review questions/objectives lend themselves to a scoping review approach.	4-5
Objectives	4	Provide an explicit statement of the questions and objectives being addressed with reference to their key elements (e.g., population or participants, concepts, and context) or other relevant key elements used to conceptualize the review questions and/or objectives.	4-5
**METHODS**			
Protocol and registration	5	Indicate whether a review protocol exists; state if and where it can be accessed (e.g., a Web address); and if available, provide registration information, including the registration number.	n/a
Eligibility criteria	6	Specify characteristics of the sources of evidence used as eligibility criteria (e.g., years considered, language, and publication status), and provide a rationale.	5
Information sources*	7	Describe all information sources in the search (e.g., databases with dates of coverage and contact with authors to identify additional sources), as well as the date the most recent search was executed.	6 (May 2020)
Search	8	Present the full electronic search strategy for at least 1 database, including any limits used, such that it could be repeated.	Table 1 and Appendix A
Selection of sources of evidence†	9	State the process for selecting sources of evidence (i.e., screening and eligibility) included in the scoping review.	6
Data charting process‡	10	Describe the methods of charting data from the included sources of evidence (e.g., calibrated forms or forms that have been tested by the team before their use, and whether data charting was done independently or in duplicate) and any processes for obtaining and confirming data from investigators.	6
Data items	11	List and define all variables for which data were sought and any assumptions and simplifications made.	5-6
Critical appraisal of individual sources of evidence§	12	If done, provide a rationale for conducting a critical appraisal of included sources of evidence; describe the methods used and how this information was used in any data synthesis (if appropriate).	n/a
Synthesis of results	13	Describe the methods of handling and summarizing the data that were charted.	6-7

For peer review only

SECTION	ITEM	PRISMA-ScR CHECKLIST ITEM	REPORTED ON PAGE #
**RESULTS**			
Selection of sources of evidence	14	Give numbers of sources of evidence screened, assessed for eligibility, and included in the review, with reasons for exclusions at each stage, ideally using a flow diagram.	6
Characteristics of sources of evidence	15	For each source of evidence, present characteristics for which data were charted and provide the citations.	8-10
Critical appraisal within sources of evidence	16	If done, present data on critical appraisal of included sources of evidence (see item 12).	n/a
Results of individual sources of evidence	17	For each included source of evidence, present the relevant data that were charted that relate to the review questions and objectives.	Tables 3, 4 and 5
Synthesis of results	18	Summarize and/or present the charting results as they relate to the review questions and objectives.	8-22
**DISCUSSION**			
Summary of evidence	19	Summarize the main results (including an overview of concepts, themes, and types of evidence available), link to the review questions and objectives, and consider the relevance to key groups.	22
Limitations	20	Discuss the limitations of the scoping review process.	22
Conclusions	21	Provide a general interpretation of the results with respect to the review questions and objectives, as well as potential implications and/or next steps.	22-25
**FUNDING**			
Funding	22	Describe sources of funding for the included sources of evidence, as well as sources of funding for the scoping review. Describe the role of the funders of the scoping review.	26

JBI = Joanna Briggs Institute; PRISMA-ScR = Preferred Reporting Items for Systematic reviews and Meta-Analyses extension for Scoping Reviews.
* Where *sources of evidence* (see second footnote) are compiled from, such as bibliographic databases, social media platforms, and Web sites.
† A more inclusive/heterogeneous term used to account for the different types of evidence or data sources (e.g., quantitative and/or qualitative research, expert opinion, and policy documents) that may be eligible in a scoping review as opposed to only studies. This is not to be confused with *information sources* (see first footnote).
‡ The frameworks by Arksey and O'Malley (6) and Levac and colleagues (7) and the JBI guidance (4, 5) refer to the process of data extraction in a scoping review as data charting.
§ The process of systematically examining research evidence to assess its validity, results, and relevance before using it to inform a decision. This term is used for items 12 and 19 instead of "risk of bias" (which is more applicable to systematic reviews of interventions) to include and acknowledge the various sources of evidence that may be used in a scoping review (e.g., quantitative and/or qualitative research, expert opinion, and policy document).

From: Tricco AC, Lillie E, Zarin W, O'Brien KK, Colquhoun H, Levac D, et al. PRISMA Extension for Scoping Reviews (PRISMAScR): Checklist and Explanation. Ann Intern Med. 2018;169:467–473. doi: 10.7326/M18-0850.

For peer review only

BMJ Open

Studying trajectories of multimorbidity: a systematic scoping review of longitudinal approaches and evidence

Journal:	*BMJ Open*
Manuscript ID	bmjopen-2020-048485.R1
Article Type:	Original research
Date Submitted by the Author:	14-Jun-2021
Complete List of Authors:	Cezard, Genevieve; University of St Andrews, Geography and Sustainable Development<br>McHale, C; University of St Andrews, School of Medicine<br>Sullivan, Frank; University of St Andrews, School of Medicine<br>Bowles, Juliana; University of St Andrews, Computer Science<br>Keenan, Katherine; University of St Andrews, Geography and Sustainable Development
**Primary Subject Heading**:	Public health
Secondary Subject Heading:	Epidemiology, Research methods, Health informatics, Global health
Keywords:	PREVENTIVE MEDICINE, PUBLIC HEALTH, INTERNAL MEDICINE, EPIDEMIOLOGY, STATISTICS & RESEARCH METHODS

SCHOLARONE™
Manuscripts

I, the Submitting Author has the right to grant and does grant on behalf of all authors of the Work (as defined in the below author licence), an exclusive licence and/or a non-exclusive licence for contributions from authors who are: i) UK Crown employees; ii) where BMJ has agreed a CC-BY licence shall apply, and/or iii) in accordance with the terms applicable for US Federal Government officers or employees acting as part of their official duties; on a worldwide, perpetual, irrevocable, royalty-free basis to BMJ Publishing Group Ltd ("BMJ") its licensees and where the relevant Journal is co-owned by BMJ to the co-owners of the Journal, to publish the Work in this journal and any other BMJ products and to exploit all rights, as set out in our licence.

The Submitting Author accepts and understands that any supply made under these terms is made by BMJ to the Submitting Author unless you are acting as an employee on behalf of your employer or a postgraduate student of an affiliated institution which is paying any applicable article publishing charge ("APC") for Open Access articles. Where the Submitting Author wishes to make the Work available on an Open Access basis (and intends to pay the relevant APC), the terms of reuse of such Open Access shall be governed by a Creative Commons licence – details of these licences and which Creative Commons licence will apply to this Work are set out in our licence referred to above.

Other than as permitted in any relevant BMJ Author's Self Archiving Policies, I confirm this Work has not been accepted for publication elsewhere, is not being considered for publication elsewhere and does not duplicate material already published. I confirm all authors consent to publication of this Work and authorise the granting of this licence.

For peer review only

Studying trajectories of multimorbidity: a systematic scoping review of longitudinal approaches and evidence.

Genevieve Cezard [1], Calum McHale [2], Frank Sullivan [2], Juliana Bowles [3], Katherine Keenan [1*]

[1] School of Geography and Sustainable Development, University of St Andrews, St Andrews, UK.

[2] School of Medicine, University of St Andrews, St Andrews, UK.

[3] School of Computer Science, University of St Andrews, St Andrews, UK.

*Corresponding author: Dr Katherine Keenan, School of Geography and Sustainable Development, University of St Andrews, Irvine Building, North Street, St Andrews, UK. KY16 8AL. Katherine.keenan@st-andrews.ac.uk

Word count: 4,604.

For peer review only

For peer review only - http://bmjopen.bmj.com/site/about/guidelines.xhtml

ABSTRACT

Objectives: Multimorbidity – the co-occurrence of at least two chronic diseases in an individual - is an important public health challenge in ageing societies. The vast majority of multimorbidity research takes a cross-sectional approach, but longitudinal approaches to understanding multimorbidity are an emerging research area, being encouraged by multiple funders. To support development in this research area, the aim of this study is to scope the methodological approaches and substantive findings of studies which have investigated longitudinal multimorbidity trajectories.

Design: We conducted a systematic search for relevant studies in four online databases (Medline, Scopus, Web of Science, and Embase) in May 2020 using pre-defined search terms and inclusion and exclusion criteria. The search was complemented by searching reference lists of relevant papers. From the selected studies we systematically extracted data on study methodology and findings, and summarised them in a narrative synthesis.

Results: We identified 35 studies investigating multimorbidity longitudinally, all published in the last decade, and predominantly in high-income countries from the Global North. Longitudinal approaches employed included constructing change variables, multilevel regression analysis (e.g. growth curve modelling), longitudinal group-based methodologies (e.g. latent class modelling), analysing disease transitions, and visualisation techniques. Commonly identified risk factors for multimorbidity onset and progression were older age, higher socio-economic and area-level deprivation, overweight, and poorer health behaviours.

Conclusion: The nascent research area employs a diverse range of longitudinal approaches that characterize accumulation and disease combinations, and to a lesser extent disease sequencing and progression. Gaps include understanding the long-term, life course determinants of different multimorbidity trajectories, and doing so across diverse populations, including those from low and middle-income countries. This can provide a detailed picture of morbidity development, with important implications from a clinical and intervention perspective.

Keywords: Multimorbidity; longitudinal; cohort; trajectories; systematic review.

For peer review only - http://bmjopen.bmj.com/site/about/guidelines.xhtml

STRENGTHS AND LIMITATIONS OF THE STUDY

- This is the first systematic review to focus on studies that take a longitudinal, rather than cross-sectional, approach to multimorbidity.
- Systematic searches of online academic databases were performed using pre-defined search terms, as well as searching of reference lists, and this is reported using PRISMA-ScR guidelines.
- For selected papers, data was double extracted using standardised proformas to aid narrative synthesis.
- Due to the heterogeneity of the studies included, their weaknesses were described in the narrative synthesis, but we did not perform quality assessment using standardised tools.

For peer review only

INTRODUCTION

The term multimorbidity is used to define the co-occurrence of multiple diseases, specifically two or more chronic conditions within the same individual [1,2]. Multimorbidity represents a huge immediate and future challenge for health care systems around the world. It is estimated that 50 million people suffer from multimorbidity in the European Union, and about one in three globally have multiple conditions [3,4]. The global prevalence of multimorbidity is expected to increase through the 21st century, as a result of increased life expectancy, population ageing, and the expansion of morbidity. For example, the prevalence of 'complex multimorbidity'- defined as four or more co-occurring chronic conditions - has been projected to increase from about 10% in 2015 to 17% in 2035 in England [5]. The implications of this for individuals and societies are stark: multimorbidity is predictive of poorer quality of life [6], greater functional decline [7], and increased mortality [8]. Management and treatment of multimorbidity also places a considerable economic and logistical burden on health services [9], which are not adapted to deal with multimorbidity, being typically organised around the single disease model.

In response to this challenge, in the last two decades there has been an explosion of (predominantly cross-sectional) research that has investigated the risk factors and patterns of multimorbidity. For example, systematic reviews have identified common clusters of diseases [10–12], which include cardiovascular and metabolic diseases, mental health conditions, and musculoskeletal disorders. Key risk factors for the multimorbidity development and severity include increasing age and poor socioeconomic status (SES) [12,13], and poor health behaviours, such as high body mass index (BMI) and smoking [14]. However, the vast majority of multimorbidity studies apply a cross-sectional approach; longitudinal approaches are scarce. To date, there are more than 70 published systematic reviews about multimorbidity, covering definitions to interventions (for example [2,15]), and none of these focuses on reviewing longitudinal studies. This study was designed to fill this clear research gap. While 'snap-shot' analyses are useful for understanding prevalence and clustering of diseases, they provide little information on multimorbidity development over time and sequencing of diseases, which have important implications from a clinical and intervention perspective. Recently, there has been a growing orientation towards longitudinal approaches by academic communities and funders such as the UK's Academy of Medical Sciences [4].

Therefore, this paper aims to gain an overview of the longitudinal approaches used in multimorbidity research, to better understand what evidence is generated from these approaches, and to identify the associated gaps.

Our research questions are:

For peer review only

1. What type and range of longitudinal methods are used to analyse multimorbidity over time within individuals?
2. What are the risk/protective factors identified to be associated with individual multimorbidity trajectories?

We used a scoping review approach to systematically review the emerging body of literature of longitudinal studies that have investigated multimorbidity trajectories. Based on a narrative synthesis focused on commonalities and differences, this review provides a methodological summary and a comprehensive synthesis of the evidence on factors affecting multimorbidity pathways.

METHODS

We review the literature on longitudinal multimorbidity studies via a scoping review approach rather than using a systematic review or meta-analytic approach [16]. Scoping reviews are adopted when the purpose of the review is to scope a nascent body of literature and appraise gaps [17,18]. In reporting, we follow the recently developed Preferred Reporting Items for Systematic Reviews and Meta-Analyses for scoping reviews (PRISMA-ScR) [19] (Appendix A).

Eligibility criteria

Inclusion and exclusion criterion were defined prior to database searches (Table 1). A primary eligibility criterion was to measure multimorbidity longitudinally within the same sample of adults using a quantitative approach; and we excluded cross-sectional or qualitative designs, reviews, meta-analyses and commentary that did not contain empirical results. Studies had to measure multimorbidity through recognised diseases /conditions or a defined multimorbidity measure such as the Charlson or Elixhauser comorbidity indices [20,21], but not solely a collection of symptoms/states (such as disability or frailty) or disease risk factors (such as obesity). Studies were required to measure change in multimorbidity between distinguishable diseases rather than progression within a single disease category (e.g. different types of cancer). We also excluded studies that examined transitions from an index disease into a secondary disease (e.g. comorbidities of diabetes). Finally, included studies were focussed on adult humans (aged 18+), and were peer-reviewed journal articles, written in English language. Our search had no restrictions on date of publication.

For peer review only - http://bmjopen.bmj.com/site/about/guidelines.xhtml

Table 1: Study inclusion/exclusion criteria for the scoping review

	Inclusion	Exclusion
**Study design**	Repeated measures designs, longitudinal quantitative studies, including retrospective and prospective cohort studies	Cross-sectional studies<br><br>Systematic reviews/meta-analyses<br><br>Qualitative studies<br><br>Expert opinion/committee reports
**Methodology**	Measure trajectories of multimorbidity longitudinally within the same individuals<br><br>Multimorbidity defined as a combination of recognised diseases/conditions (e.g. self-report or ICD 9/ICD 10 codes)<br><br>Trajectories defined as change or accumulation in number of distinguishable diseases	Different cohorts/samples used across longitudinal study timeline<br><br>Multimorbidity defined as combination of symptoms or pre-disease conditions, i.e. not defined ICD-10 diseases (e.g. pre-disease, frailty, disability, quality of life)<br><br>Transitions or trajectories within a single disease (e.g. dementia), or from one disease into another (e.g. cancer progression)
**Population**	Adult humans (18+ years)	Infants, children or adolescents (<18 years)<br><br>Animal research
**Publication**	Peer-reviewed journal articles<br><br>Accessible in English	Grey literature<br><br>Not accessible in English

For peer review only - http://bmjopen.bmj.com/site/about/guidelines.xhtml

Search strategy

Four online databases were searched: Medline, Scopus, Web of Science, and Embase. Initially, scoping searches were conducted within each database, with relevant terms such as 'multimorbidity', 'disease trajectory', and 'longitudinal'. These scoping searches allowed the identification of additional relevant search terms and, where appropriate, MeSH headings, in order to develop and refine the final search strategy (Table 2).

Table 2. Summary of search strategy

Search no.	Search terms
#1	Multimorbidity (multimorbid*; multi-morbid*) OR Comorbidity (comorbid*; co-morbidi*) OR Cooccurrence (cooccur*; co-occur*)
#2	disease OR condition OR illness AND cluster* OR trajectory (trajector*) OR cascade* OR accumulation (accumulat*) OR combination* OR sequence (sequenc*) OR transition*
#3	cohort* OR longitudinal* OR prospective*
#4	#1 AND #2 AND #3
#5	#4 AND NOT cell* OR gene OR genes OR bacteria* OR DNA OR COVID-19 (covid*)

The final search was a combination of three search elements: first, the concept of multimorbidity, second the methodological approach of disease trajectories, and third, longitudinal study design. These search terms initially returned a large number of irrelevant references, focusing on cellular medicine, genetics, and Covid-19, so we added an additional condition to exclude these. We also refined the search results to include English language, adult humans, and peer-reviewed journal articles only. All searches were conducted in May 2020. The full search syntax is included in Appendix B. We identified additional relevant papers through recommendations from co-authors and external collaborators. The database search results were searched for these additional papers, and if they were not identified in the database searches, they were included as *'identified through other sources'* and were subject to the same screening procedure as papers identified through database searches.

Screening and study selection

After de-duplication, articles were screened for eligibility by title, abstract and finally full text (work shared between GC, CM, and KK) using Endnote and pre-defined groups for exclusion reasons and

For peer review only

For peer review only - http://bmjopen.bmj.com/site/about/guidelines.xhtml

inclusion. At abstract and full text stages, a double screening process was used to minimise evidence selection bias [22], meaning two co-authors blindly and independently reviewed the study for inclusion. Any disagreements were resolved through discussion and consensus. The reference lists of the selected studies were screened to identify any relevant studies that may have been missed in the main search, and any newly identified articles were subject to the same screening and data extraction processes.

Data extraction and synthesis

Three authors (GC, CM, KK) extracted and double-extracted information from selected articles. We extracted information on study and sample characteristics, including the title, authors and publication year, study setting, data source used, information on the study population (e.g. inclusion and exclusion criteria, sample size and age), and follow-up duration. We also extracted study objectives, information on multimorbidity conceptualisation and measures, and methodological and analytical approaches, focussing on those specifically used for the analysis of multimorbidity trajectories. Finally, we extracted the key findings, and limitations reported in each study in relation to generalisability, accuracy, comprehensiveness, methodology, and interpretation.

To develop the narrative synthesis, we analysed and summarised the patterns in the extracted data, investigated the similarities and differences between studies, and examined bias and limitations to identify knowledge gaps and the strengths and weaknesses of methodological approaches.

Ethics Approval

This is a review of already published material, therefore no ethics approval needed.

Patient and Public Involvement

No patient involved.

RESULTS

Study selection

Figure 1 depicts the study selection process. Database searches returned 11,420 articles and 9 additional papers were identified from other sources. Of the combined 11,429 papers, 4,705 were duplicate references and removed. Of the remaining 6,724 papers, 6,315 were removed during title screening and a further 360 papers during abstract screening. The most common reasons for exclusion were studies which did not focus on multimorbidity longitudinally (e.g. trajectories were followed within a single disease) and study design not being longitudinal (e.g. cross-sectional analysis). The remaining 49 papers went through full-text screening and 19 subsequently were removed during this process. Searching the reference lists of the remaining 30 papers identified

For peer review only - http://bmjopen.bmj.com/site/about/guidelines.xhtml

another 11 potentially relevant papers. After screening these 11 papers, 6 were excluded leaving 5 additional papers for inclusion. In total, 35 papers were selected for further data extraction.

[Fig 1 about here]

For peer review only

For peer review only - http://bmjopen.bmj.com/site/about/guidelines.xhtml

Characteristics of the selected studies

Table 3 summarises the study characteristics. All articles were published since 2011, and were primarily based on data from European countries (N=16) [23–38], Australia and North America (N=14) [39–52], and high-income Asian countries South Korea, Taiwan, and Singapore (N=4) [53–56]. Apart from one study using Chinese data [57], none related to low- and middle-income settings. Sample characteristics varied widely. For example, sample size ranged from 756 in a survey of older participants [45] to 6.2 million in a nationwide study using Danish register data [38], and the length of follow-up periods ranged between 2 and 20 years. Most studies had age restrictions, with about half focused on older populations (50 years+), and one study focused on the very old (80 years plus) [36]. Most samples included both males and females apart from two studies including males only [26,37] and three studies including females only [42,47,51]. Furthermore, three studies focused on U.S. veterans, a predominantly male population [39,44,48]. The data sources used were a combination of administrative data, including primary or secondary care records, disease registries, and health insurance data (24 studies), and survey data (18 studies). There were common datasets used across studies, such as the Swedish National study on Aging and Care in Kungsholmen (SNAC-K) [27,31–33]. In seven studies, survey data was combined with administrative data sources [27,28,30–33,43]. In five survey-based studies, questionnaire data was supplemented by medical examination records, or cognitive or laboratory tests [29,34,45,52,57]. Informed consent of participants was mentioned in 10 of the 18 studies using survey data.

For peer review only - http://bmjopen.bmj.com/site/about/guidelines.xhtml

Table 3: Characteristics of the selected studies

Study, year	Country	Sample size	Baseline sample age	Follow-up period	Data source	Number of conditions included	Disease identification strategy
Alaeddini et al. (2017)	USA	N=601,805	Adults	14 years	Administrative data: inpatient and outpatient data	4	ICD-9-CM codes
Ashworth et al. (2019)	UK	N=332,353	18 years+	14.5 years	Administrative data: primary care records	12	Read codes in UK primary care (based on the Quality and Outcome Framework)
Beck et al. (2016)	Denmark	N≈120,000	All ages	18.5 years	Administrative data: inpatient and outpatient data, and emergency visits	All diseases taken at three level ICD 10 code	ICD 10 codes (A41 specified for sepsis)
Calderon-Larranaga et al. (2018)	Sweden	N=2,387	60 years+	3 to 6 years	Survey with interviews, clinical examinations, cognitive tests, and laboratory tests combined with and administrative data: National Patient Register, death	918 diseases grouped into 60 categories	ICD10 codes
Calderon-Larranaga et al. (2019)	Sweden	N=2,293	60 years+	9 years	Survey with interviews, clinical examinations, cognitive tests, and laboratory tests combined with and administrative data: National Patient Register, death	918 diseases grouped into 60 categories	ICD10 codes
Canizares et al. (2017)	Canada	N=10,186	20 -69 years	18 years	Survey data	17	Self-reported
Chang et al. (2011)	Taiwan	N=147,892	All ages	3 years	Administrative data: national insurance claims data including inpatient, outpatient, ambulatory care, dental care, and pharmacy expenditure	Not specified	ICD-9-CM codes
Dekhtyar et al. (2019)	Sweden	N=2,589	60 years+	9 years	Survey with interviews, clinical examinations, cognitive tests, and laboratory tests combined with and administrative data: National Patient Register, death	918 diseases grouped into 60 categories	ICD10 codes
Fabbri et al. (2015)	Italy	N=1,018	60 years+	9 years	Survey data with clinical examinations and laboratory tests	15	Self-reported medical history, medication use, medical documentation, and clinical examination.
Fabbri et al. (2016)	USA	N=756	65 years+	9 years	Survey data with clinical examinations and laboratory tests	13	Self-reported, laboratory tests, and clinical examination
Faruqui et al. (2018)	USA	N=257,633	18 years+	5 years	Administrative data: inpatient and outpatient data	5	ICD-9-CM codes
Fraccaro et al. (2016)	UK	N=287,459	18 years+	9.5 years	Administrative data: primary and secondary care, death, and out-migration data	22	Read codes in UK primary care data (based on validated codes by Khan et al. (2010))
Freisling et al. (2020)	Seven European countries (Denmark, Germany, Italy, the Netherlands, Spain, Sweden, and the UK)	N = 291,778	35-70 years	16 years	Administrative data (primary and secondary care, disease registry, health insurance, medication use, and death data) and survey (questionnaire at baseline)	3	Combination of ICD10 codes and disease registry
Gellert et al. (2018)	Germany	N=1,398	80 years+	6 years	Administrative data: ambulatory, hospital, and long-term care	30	ICD10 codes
Hanson et al. (2015)	USA	N=41,158	65-84 years	18 years	Administrative data: UPDB, death, and Medicare claims	17	Diseases are identified following the SEER-Medicare Comorbidity macro from Medicare data in 1992-2009

For peer review only - http://bmjopen.bmj.com/site/about/guidelines.xhtml

Hiyoshi et al. (2017)	Sweden	N=5,218	44-51 years	5 years	Administrative data: Cancer register and Patient register with inpatient and outpatient data	18	Swedish version of ICD-9 and 10
Hsu (2015)	Taiwan	N=2,584	60 years+	14 years	Survey data	15 conditions grouped into 6 disease types	Self-reported
Jackson et al. (2015)	Australia	N=4,865	47-52 years	12 years	Survey data	18	Self-reported
Jensen et al. (2014)	Denmark	N=6.2 million	All ages	14.9 years	Administrative data: inpatient, outpatient, and emergency visits	All diseases taken at three level ICD 10 code	ICD10 codes
Kim et al. (2018)	South Korea	N=121,733	60 years+	11 years	Administrative data: claims data from private healthcare providers.	20	Claims data
Lappenschaar et al. (2013)	The Netherlands	N=182,396	35 years+	9 years	Administrative data: primary care data.	6	International Classification of Primary Care (ICPC) codes, completed with laboratory tests and medication information.
Lindhagen et al. (2015)	Sweden	N=20,237	Adults	10 years	Administrative data: cancer and population registers.	17	ICD codes from Patient and Cancer Registries
Perez et al. (2020)	Sweden	N=2,596	60 years+	6 years	Survey with interviews, clinical examinations, cognitive tests, and laboratory tests combined with and administrative data: National Patient Register, death	918 diseases grouped into 60 categories	ICD10 codes
Pugh et al. (2016)	USA	N=164,933 (derivation) N=146,051 (validation)	Adults	3 years	Administrative data: inpatient, outpatient, and medications data	Not specified	ICD-9-CM codes
Quinones et al. (2011)	USA	N=17,517	51 years+	11 years	Survey data	7	Self-reported
Quinones et al. (2019)	USA	N=8,331	51- 55 years	16 years	Survey data	7	Self-reported
Rocca et al. (2016)	USA	N=3,306	Adults < 50	10-19 years	Administrative data: medical records.	18	ICD-9 codes
Ruel et al. (2014)	Australia	N=1,854	18 years+	6-10 years	Survey (with telephone interview, self-completed questionnaire, and clinic biomedical assessment including blood sample)	8	Self-reported and biomedical measurements
Ruel et al. (2014)	China	N=1,020	20 years+	5 years	Survey (with laboratory tests)	11	Self-reported, blood samples, and clinical examinations
Ryan et al. (2018)	Republic of Ireland	N=4,502	50 years+	2 years	Survey (with health assessment in a health centre)	30 diseases grouped into 16 categories	Self-reported and clinical examination
Siriwardhana et al. (2018)	USA (Hawaii)	N=22,930	65 years+	5 years	Administrative data (health insurance data)	3	ICD9 codes
Strauss et al. (2014)	UK	N=24,615 (Phase I) N=4,532 (Phase II)	50 years+	3 years	Administrative data (primary care records for CiPCA) linked to Questionnaire data (NorStOP)	42	Read codes in UK primary care
Xu et al. (2018)	Australia	N=11,941	45-50 years	20 years	Survey data (self-reported)	3	Self-reported
Zeng et al. (2014)	USA (Colorado)	N=961 (Primary cohort) N=13,163 (secondary cohort)	65 years+	10 years	Administrative data (electronic health records including inpatient admission and emergency visits, and death) and survey	17	ICD-9-CM codes based on Quan et al. (2005) algorithm
Zhu et al. (2018)	Singapore	N≈700,000	All ages	7 years	Administrative data (electronic health records and deaths)	6	Not specified

For peer review only

For peer review only - http://bmjopen.bmj.com/site/about/guidelines.xhtml

Methods of disease and multimorbidity ascertainment

Studies based on administrative data relied on clinician-diagnosed diseases, often using standardised diagnosis codes, such as the International Classification of Diseases i.e. ICD-9, ICD-9-CM, or ICD-10. All survey-based studies used participant self-report for disease identification. Studies that combined survey data with other sources ascertained disease status mostly through clinician-diagnosis [27,28,30–33,43] but some supplemented with laboratory and cognitive tests [29,34,45,52,57]. The number of diseases which were considered to contribute to the measure of multimorbidity varied widely, ranging from three [30,41,42] to a very large number based on three levels of ICD-10 codes [24,38]. Studies using survey data used a narrower range of diseases than those drawn from administrative data. The precise list of diseases was never uniform between studies (see Appendix C for full details), but the rationale for choosing them was usually described. For example, included diseases with high prevalence and risk of disability and mortality [34,45], or that were assessed/validated by clinicians [27,28,31–33]. Some used lists based on the Charlson and Elixhauser multimorbidity indices [26,35–37,43,46,56] but these were sometimes augmented with extra conditions [37] or reduced due to data sensitivity restrictions [35].

Approaches to the measurement of multimorbidity trajectories

To develop longitudinal measures of multimorbidity, studies tended to take one of two broad approaches. The most common was that repeated measures of multimorbidity status over time were measured for each individual. This mainly involved constructing unweighted or weighted counts of diseases at regular intervals for each individual, thus conceptualising multimorbidity as a continuum (for example [52,57]), although a few still used a binary measure of two or more chronic conditions [29,40]. The second broad approach explored disease transitions [24–26,30,38,39,41,42,44,55]. Only one study explored the order of disease occurrence [23].

Types of methodology

Breaking this down further, we identified five broad analytical approaches: constructed variables of multimorbidity change, multilevel regression modelling, transition and data mining methodologies, visual approaches (articles summarised in Table 4) and longitudinal group-based methodologies (articles summarised in Table 5). Note that some studies employed more than one type of approach (e.g. Xu et al., 2018 [42]). In the first approach, four articles created variables of multimorbidity change [29,35,53,57]. In one study, intra-individual change in Charlson Comorbidity Index (CCI) between baseline and later time points was used [35], and in another, transitions to two or more conditions, or by acquisition of additional conditions [29]. Two studies used simple methods to

For peer review only - http://bmjopen.bmj.com/site/about/guidelines.xhtml

construct morbidity trajectory groups (e.g. 'constant high', 'constant medium', 'constant low') [53] and disease transition stages (e.g. 'healthy', 'healthy to a single chronic disease') [57]. After creating these categorical dependent variables the authors used them in regression analysis to assess their association with health expenditures [53], diet [57], and physical activity and functioning [29].

The next approach, employed by 14 studies (Table 4) [27,31–36,40,42,43,45,49,50,52] was multilevel regression modelling (variously referred to as random effects models, growth curve models, hierarchical linear models or multilevel models). These studies analyse repeated measures of multimorbidity within each individual, considering this as a ‘trajectory’ or ‘growth curve’. The dependent variable was typically a count of diseases or a multimorbidity index measured repeatedly, and the coefficients assessed a change in this over time, many including random effects for both the intercept and slope. One study used the regression estimates (i.e. intercept and slope coefficients for multimorbidity) to create categories capturing the pace of multimorbidity e.g. 'rapidly accumulating' and 'slowly accumulating', which were used for further modelling [31]. Some of these studies also investigated whether certain covariates such as biomarkers [27,34], socio-demographics and life experiences [33] affected the pace of change in multimorbidity by including an interaction term between time and the respective covariates.

For peer review only

Table 4. Methods of studies taking four analytical approaches (multimorbidity change variables, regression, transition modelling and visual approaches)

Study, year	Main study outcome	Statistical methodology used to analyse multimorbidity longitudinally
**Approach 1: Constructed variables of multimorbidity change (N=4)**		
Chang et al. (2011)	Prospective medical use and expenditures	**Morbidity trajectory groups**: constant high, constant medium, constant low, decreasing, increasing, and erratic.
Fraccaro et al. (2016)	Time to death from any cause	**Change in comorbidities:** difference between baseline Charlson Comorbidity Index (CCI) and 1, 5, 10-year follow-up CCI scores as a proportion of mortality rate.
Ruel et al. (2014)	Six chronic disease transition stages groups	**Groups of chronic disease transition stages** (healthy, healthy to a single chronic disease, stable with a single chronic disease, healthy to multimorbidity, stable multimorbidity, and increasing multimorbidity)
Ryan et al. (2018)	Development/worsening of multimorbidity	**Constructed measures of multimorbidity change** (from zero or one condition at baseline to two or more conditions) and **of worsening of multimorbidity** (from multimorbidity at baseline to additional conditions at follow-up)
**Approach 2: Regression-based approaches (N=14)**		
Calderon-Larranaga et al. (2018)	Disability (IADL[1] count)	**Linear mixed models[2]** estimated the rate of multimorbidity accumulation. Predicted slopes are examined by quartile and further dichotomised into "rapidly accumulating" (upper quartile) and "slowly accumulating" (three lower quartile).
Calderon-Larranaga et al. (2019)	Level of multimorbidity (count of chronic conditions) and disability (IADL count)	**Linear mixed models** assessed the association between baseline level of psychological factors and multimorbidity and disability over time. The interaction terms between time and each of the psychological factors were included as a fixed effect.
Canizares et al. (2017)	Multimorbidity (binary- 2 or more conditions)	**Multilevel logistic growth modelling** was used to examine the age, period, and cohort effects on multimorbidity. Observations nested in individuals and age and birth cohort entered as fixed effects.
Dekhtyar et al. (2019)	Level of multimorbidity (count of chronic conditions)	**Linear mixed models** assessed the association between life experiences and the speed of multimorbidity accumulation over time. Interaction terms between time and life experiences included as fixed effects.
Fabbri et al. (2015)	Level of multimorbidity (count of chronic conditions)	**Linear mixed models** assessed the association between baseline age, disease status, and biomarkers with the number of diseases over follow-up. The study also tested whether increased IL-6 over time would predict steeper increase in multimorbidity over time, independent of baseline IL-6.
Fabbri et al. (2016)	Standardized neurocognitive tests evaluating cognitive function	**Linear mixed models** were used to estimate rate of change in multimorbidity (count of diseases). The individual slopes of multimorbidity rise were dichotomized into faster accumulation (upper quartile) and the rest (lower 3 quartiles).
Fraccaro et al. (2016)	All-cause mortality	Multimorbidity change was measured by differences between baseline Charlson Comorbidity Index (CCI) and 1, 5, 10-year follow-up CCI scores as a proportion of mortality rate. **Survival analysis (Cox regression)** estimated mortality rates as a function of age, gender and CCI scores (fixed and time-varying).
Gellert et al. (2018)	Level of multimorbidity (count of comorbidities- based on Elixhauser)	**Linear mixed models** (random intercepts and slopes) estimated differential increase in the number of comorbidities over 25 calendar quarters prior to death in centenarian, nonagenarian, and octogenarian cohorts.
Perez et al. (2020)	Level of multimorbidity (count of chronic conditions)	**Linear mixed models** (random intercepts and slopes) were employed to analyse the association between baseline tGSH levels and level of multimorbidity.
Quinones et al. (2011)	Level of multimorbidity (count of chronic conditions)	**Linear mixed models** (random intercepts and slopes) analysed ethnic variations in level of multimorbidity.
Quinones et al. (2019)	Level of multimorbidity (count of chronic conditions)	**Negative binomial generalized estimating equation (GEE) models** with a first-order autoregressive covariance structure were used to assess the relationship between chronic disease accumulation and race/ethnicity.
Ruel et al. (2014)	Count of chronic conditions + incidence of multimorbidity	**Multinomial logistic regression** estimated the count and individual proportion of chronic diseases in those with no or one chronic disease at baseline.

For peer review only

Xu et al. (2018)	Cumulative incidence of three conditions (diabetes, heart disease and stroke)	**Repeated measures logistic regression** using **generalized estimating equations (GEE)** were used to identify risk factors for developing three conditions and their combinations. **Generalised linear mixed models** were used to estimate the associations between predictors and the progression to multimorbidity.
Zeng et al. (2014)	Self-reported health, number of primary care visits, inpatient admissions, emergency department visits, and mortality	**Linear mixed models** (random intercepts and slopes) estimated the individual trajectory of Charlson Comorbidity Index (CCI) over time (up to 10 years), which was used as an independent variable in a subsequent linear regression model for the health outcomes.
**Approach 3: Transition and disease progression modelling (N=9)**		
Alaeddini et al. (2017)	Clusters of disease transition considering four conditions (hypertension, depression, PTSD and back pain)	Disease transitions were modelled using **Markov chain models with a transition matrix, placed in a Latent Regression Markov Mixture Model** to incorporate subject–specific covariates (e.g., age, sex, race/ethnicity, etc.). Markov Clustering algorithm used to identify patterns of disease progression.
Beck et al. (2016)	30-day mortality in patients with sepsis	Data driven method combining **temporal directed pairs** for identification of disease trajectories based on the method developed by Jensen (2014)
Freisling et al. (2020)	Transition to Cancer-cardiometabolic multimorbidity	Non-Markovian **multi-state modelling** for transitions to cancer, CVD, Type 2 Diabetes, and subsequently to MM using cox proportional hazards.
Faruqui et al. (2018)	Development of 5 specific conditions	Comparison of several methods: **unsupervised Bayesian network, multivariate regression and latent regression Markov mixture modelling. Longest Path Algorithm (LPA)** from the Bayesian network was used to identify the most probable sequence from/to a specific disease.
Jensen et al. (2014)	Disease trajectories	**Temporal correlation analysis** i.e. strength of correlation between the pair of diseases (relative risk >1) for over a million pairs where disease 2 (D2) occurs within 5 years of disease 1 (D1) and based on directionality (whether D1->D2 occurs more often than D2->D1, binomial tests).**Disease trajectories combining pairs** with overlapping diagnosis, into three or more diseases.
Lindhagen et al. (2015)	Mortality; change and scale of CCI change	A **state transition model** in discrete time steps to estimate changes in Charlson Comorbidity Index (CCI). Transition probabilities were estimated using l**ogistic/Poisson regression models** for vital status, and CCI changes. **Simulation models** estimated changes in CCI with their confidence intervals.
Lappenschaar et al. (2013)	Cumulative incidence and combinations of six cardiovascular diseases	**Multilevel temporal Bayesian Networks** were used to model the patient's disease status at baseline, and 3-5 years after. The variance induced by the urbanization level, age, and gender, in the multilevel model was explained using Markov Chain Monte Carlo simulation.
Siriwardhana et al. (2018)	Disease state probability and transition probability from a single disease state to a multiple disease state.	**Acyclic multistate model** to define an interconnected progressive chronic disease system for the elderly population. **Aalen and Johansen estimator** (a nonparametric technique) to estimate marginal state occupational probabilities.
Zhu et al. (2018)	Disease progression states and the absorbing state, death. Life-years lost to a specific condition and cumulative lifetime risk of certain conditions	**A disease progression network was constructed** based on the real cohort. One-year progression from state A to B is calculated by counting the number of people who are in state A the previous year and in state B the following year. **Microsimulation** is used to calculate life-years-lost and lifetime risk of particular states.
**Approach 4. Visualisation methods (N=3)**		
Ashworth et al. (2019)	Level of multimorbidity (count of chronic conditions)	**Alluvial plots** based on date of onset of each long-term condition, tabulated as first, second and third to visualise the acquisition sequence
Rocca et al. (2016)	Level of multimorbidity (count of chronic conditions)	The accumulation of multimorbidity was represented graphically using **Aalen-Johansen curves** (a multistate generalization of cumulative incidence curves; unadjusted curves considering all 18 conditions equally).
Xu et al. (2018)	Cumulative incidence of three conditions	**Sankey diagram** was constructed to characterize the dynamic changes of different combinations of the three conditions over time.

[1] IADL = limitations in instrumental activities of daily living. [2] Linear mixed models here refer to any multilevel model for repeated measures over time for each individual, incorporating various labels- mixed linear model, hierarchical models, growth curve model, etc.

For peer review only - http://bmjopen.bmj.com/site/about/guidelines.xhtml

The next approach, employed by nine studies [24–26,30,38,39,41,44,55], focussed on modelling transitions between specific disease states. Six of the studies focused on a limited number of diseases, to make the analysis feasible [25,30,39,41,44,55]. Some studies used principles of state transition modelling, either using Markov principles [44], acyclic multistate models [41] or state transition modelling [26,30]. Another two studies employed Bayesian techniques including a multilevel temporal Bayesian network [25] and a longest path algorithm to identify the most probable sequence from/to a specific disease following an unsupervised multi-level temporal Bayesian network analysis [39]. One paper derived a disease progression network from real data, and used this for further microsimulation [55]. Finally, two studies used a data-driven approach to create 'temporal disease trajectories' by combining significant temporal directed pairs from all disease pairs possible [24,38]. Transition analysis also enabled the identification of longitudinal clusters [44].

Three studies [23,42,51] used visual methods to describe disease sequences, or multimorbidity acquisition sequences. Ashworth et al. (2019) [23] used alluvial plots to illustrate multimorbidity acquisition sequences based on date of disease onset, and although useful to understand the order of diseases (co-)occurrence, the visualisations are unable to account for the pace of multimorbidity progression. Aalen-Johansen curves (a multistate generalization of cumulative incidence curves) were used to represent the accumulation of multimorbidity graphically [51] and the Sankey diagram to show the longitudinal progression and transitions to each disease and disease combinations [42].

The final approach, employed by seven studies [28,37,46–48,54,56], was to construct meaningful categories of longitudinal multimorbidity patterns (and associate these with other covariates (summarised in Table 5). Methodologies included latent class analysis, latent class growth analysis, growth mixture modelling, or group-based trajectory modelling, and typically identified between four to six groups of distinct longitudinal multimorbidity patterns. Two studies took an associative approach to explore which specific diseases cluster longitudinally [48,54]. For example, Hsu (2015) [54] found four trajectory groups: 'low risk', 'cardiovascular risk only', 'gastrointestinal and chronic non-specific lung disease', and 'multiple risks'. The other five studies focused on stages of accumulation. For example, Hiyoshi et al. (2017) [37] found four trajectory groups ranging from 'a constant low trajectory' to 'a high start and a slow increase trajectory'. Generally, these clusters incorporated data on the initial level of multimorbidity, and accumulation pattern over time, and nearly all showed increased accumulation (the exception being Kim et al. (2018) [56] which identified some groups with decreasing morbidities).

For peer review only - http://bmjopen.bmj.com/site/about/guidelines.xhtml

Table 5. Methods of studies investigating multimorbidity trajectory groups

Study, year	Statistical approach used	Trajectory groups identified
Hanson et al. (2015)	**Finite mixture modelling** (Proc TRAJ in SAS) was used, with a zero-inflated distribution. Optimal number of groups determined by Bayesian Information Criterion (BIC).	Six groups: 'robust' (no conditions), 'initiates' (none at baseline , increase over time), 'slow initiates' (some at baseline and gradual increase over time), 'accelerated initiates' (none at baseline and quick increase followed by deceleration), 'chronic low' (steady comorbidity over time), 'ailing' (moderate levels of comorbidity at baseline and steady increase over time), 'frail' (high comorbidity at baseline, remaining high over time).
Hiyoshi et al. (2017)	**Group-based trajectory modelling**, using a zero-inflated distribution. Optimal number of groups determined using BIC.	Four groups identified: 'a constant low trajectory', 'a low start and an acute increase trajectory', 'medium start and a slow increase trajectory', 'a high start and a slow increase trajectory'.
Hsu (2015)	**Multiple group-based trajectory model** (Proc TRAJ in SAS) Morbidity was set to follow a logistic model. The optimal group number was determined using the BIC and parsimony principle.	Four chronic disease trajectories were identified: 'low risk', 'cardiovascular risk only', 'gastrointestinal and chronic non-specific lung disease', and 'multiple risks'
Jackson et al. (2015)	**Latent class growth analysis (LCGA)** in Mplus; optimal group number determined using BIC.	Five groups identified: 'no morbidity, constant', 'low morbidity, constant', 'moderate morbidity, constant', 'no morbidity, increasing', and 'low morbidity, increasing'.
Kim et al. (2018)	**Growth mixture modelling (GMM)** in SAS was used; optimal group number determined using BIC.	Five groups identified: 'consistently low', 'increased', 'decreased (low)', 'decreased (high)', and 'consistently high'.
Pugh et al. (2016)	**Latent Class Analysis (LCA)**, based on the distribution of repeated measures in the 20 binary diagnosis outcomes.	Five groups identified for both men and women: 'Healthy', 'Chronic Disease', 'Mental Health', 'Pain' and 'Polytrauma Clinical Triad (PCT: pain, mental health and traumatic brain injury'. Two additional classes found in men were 'Minor Chronic' and 'PCT with chronic disease'.
Strauss et al. (2014)	**LCGA** in Mplus, Optimal group number determined by iterative modelling and BIC values and likelihood ratio test. Morbidity counts were assumed to be Poisson distributed. Quadratic growth curves were applied for all groups identified within the LCGA models.	Five groups identified and validated: no recorded chronic problems', developed a first chronic morbidity over 3 years', 'a developing multimorbidity group', 'increasing number of chronic morbidities', and 'a multi-chronic group with many chronic morbidities'.

For peer review only

For peer review only - http://bmjopen.bmj.com/site/about/guidelines.xhtml

Results of the studies: outcomes and risk factors

Prediction of other health outcomes

Seven of the studies used multimorbidity trajectories to predict subsequent health outcomes [31,35,43,45,53,55,56], including self-reported health, cognitive ability, disability, medical utilisation, and mortality (Table 6). Among older adults, results showed that an increase in multimorbidity over 10 years was associated with worse reported health [43], and that those who developed multimorbidity faster had greater risk of disability [31]. In one study, changes in multimorbidity were found to be more predictive of mortality than baseline multimorbidity [35]. By contrast, another study confirmed that a change in CCI predicts mortality but not necessarily better than a cross-sectional estimate of multimorbidity [43]. Zhu et al. (2018) found that earlier development of chronic conditions and earlier complications incur greater life-years lost. Finally, multimorbidity accumulation (as a marker of physical health deterioration) predicted faster decline in verbal fluency in older adults without cognitive impairment or dementia [45].

For peer review only

For peer review only - http://bmjopen.bmj.com/site/about/guidelines.xhtml

Table 6. Summary of association analysis for health outcomes related to longitudinal multimorbidity trajectories

Study, year	Outcome investigated	Findings of association analysis
Calderon-Larranaga et al. (2018)	Disability	The speed of multimorbidity is a strong predictor for disability in older adults, even when accounting for baseline number of chronic conditions
Chang et al. (2011)	Medical utilisation	Morbidity strata predicted medical utilization as usefully as more complex risk adjusters.
Fraccaro et al. (2016)	Mortality	Change over time of Charlson Comorbidity Index (CCI) was a stronger predictor of mortality than baseline CCI
Fabbri et al. (2016)	Standardized neurocognitive tests	Accumulation of multimorbidity was associated with faster decline in verbal fluency but seems to have no effect on memory decline, in older adults without mild cognitive impairment or dementia.
Kim et al. (2018)	Mortality	The 'consistently high' multimorbidity trajectory group had the highest risk of mortality at 1-, 3- and 5-year follow-ups.
Zeng et al. (2014)	Self-reported health, number of primary care visits, inpatient and emergency admissions, mortality	Growth curve models gave marginally better fitting models for the outcomes of SRH, but mortality and inpatient status was best predicted by multimorbidity snapshot prevalence the year before the survey.
Zhu et al. (2018)	Life expectancy	Diabetes, plus hypertension plus complications reduced life expectancy the most. The earlier the onset of multimorbidity, the greater the reduction in life.

For peer review only

For peer review only - http://bmjopen.bmj.com/site/about/guidelines.xhtml

Risk factors for multimorbidity

Nineteen of the selected articles [23,25,27–30,32–34,37,40–42,46,47,49,50,54,57] investigated risk factors of multimorbidity trajectories (Table 7). Increasing age, although often accounted for in analyses, emerged as a dominant risk factor for acquisition, worsening or progression of multimorbidity [29,42]. As expected, younger age groups were more likely to belong to a non-chronic healthier cluster [28]. However, trajectories starting with depression were more prevalent in younger individuals [23]. Younger cohorts were also found to be more likely to develop multimorbidity and to do so at a younger age [40]. A few studies reported gender differences, with conflicting results. While one study found that those in the 'multiple risks' group were more likely to be female [54], another two studies found that men were more likely to transition between disease states than women [30,41].

Four studies investigated ethnic variations [23,41,49,50]. In two US studies, compared to non-Hispanic whites, Black Americans had a higher rate of multimorbidity at baseline along with a slower rate of disease accumulation over time while Hispanic participants tended to start with fewer diseases and increase more rapidly [49,50]. Different ethnicities also had different disease transition patterns. In the US, white individuals were more likely to transition from ischaemic heart disease to death, while Asian and Native Hawaiian and Pacific Islander individuals were more likely to transition from diabetes to diabetes plus chronic kidney disease [41]. In the UK, disease specific sequences also differed by ethnicity: For example, the white ethnic group was dominated by depression as a starting point, while diabetes was the most common starting point in the black ethnic group [23].

The studies also explored a range of socio-demographic determinants including area-based deprivation, education, occupation, income, and marital status. Results largely confirm those found with cross-sectional analyses, with lower socioeconomic status associated with worse multimorbidity trajectories. For example, lower levels of education were associated with higher rate of multimorbidity accumulation [33,42] or worse multimorbidity trajectories [47]. People living in more deprived areas were more likely to be in an evolving or multi-chronic multimorbidity cluster [28] and to have trajectories with diabetes and depression as the most common starting point [23].

For peer review only

Table 7. Summary of association analysis for risk factors related to multimorbidity trajectories

Study, year	Risk factors	Findings of association analysis
Ashworth et al. (2019)	Age, ethnicity, deprivation	Trajectories varied by age, ethnicity, and deprivation. Depression as a starting point was more common in younger, more deprived, and white ethnic group.
Calderon-Larranaga et al. (2019)	Attitude towards life and health	Better attitudes towards life and health were associated with slower multimorbidity development, independent of demographic, clinical, social, personality, and lifestyle factors.
Canizares et al. (2017)	Birth cohort	In each succeeding cohort, multimorbidity rates was higher and multimorbidity emerged earlier. Differences persisted independently of the risk factors for multimorbidity and period effect.
Dekhtyar et al. (2019)	Elementary education (early adulthood), lifelong active occupation (mid-adulthood), social network (later life)	Adults over 60 years old with higher than elementary education, lifelong active occupations and richer social networks had slower multimorbidity accumulation. The association between childhood circumstances and multimorbidity accumulation was attenuated by subsequent (mid- and late-) life experiences. Rich social networks reduced the speed of disease accumulation irrespective of lifelong job stress and level of education.
Fabbri et al. (2015)	Biomarkers: IL-6, IL-1ra, TNF-α receptor II, and DHEAS (as a marker of chronic inflammation and system dysregulation)	Multimorbidity development with age was not linear, and significantly accelerated at older ages. Higher IL-6, IL-1ra, and TNF-α receptor II and low DHEAS were associated with higher multimorbidity at baseline, independent of age, sex, BMI, and education. Higher IL-6 and steeper increase in IL-6 predicted an accelerated rise in multimorbidity over 9 years of follow-up.
Freisling et al. (2020)	Sex, age, healthy lifestyle (healthy lifestyle index): diet (Mediterranean Diet Score), alcohol, smoking status and duration, physical activity (Cambridge index), and BMI. Education, menopausal status, use of hormones for post-menopausal women.	Healthy lifestyle habits were strongly associated with lower incident multimorbidity of cancer and cardiometabolic diseases<br>The risk of transitioning to multimorbidity after having developed a first of the 3 chronic diseases was higher in men than in women.
Hanson et al. (2015)	Parity, timing of childbearing, birth outcomes of offspring	High parity, early childbearing, and adverse offspring birth outcomes are associated with particular later-life comorbidity patterns and trajectories, when controlling for early-life conditions (age at parental death, childhood socioeconomic status, familial excess longevity, religious participation)
Hiyoshi et al. (2017)	Income, marital status	Income, and physical, cognitive, and psychological function were associated with trajectory group membership in unadjusted analysis but not in fully adjusted analysis.
Hsu (2015)	Gender, education, physical function, depressive symptoms, life satisfaction, number of health examination, smoking and drinking	Those in the 'multiple risks' group were more likely to be female, less educated, with more physical function difficulties, more depressive symptoms, lower life satisfaction, more health examinations, and not to smoke or drink. Members in the 'CVD risk only' and 'multiple risks' groups were more likely to have physical function difficulties and depressive symptoms.
Jackson et al. (2015)	Overweight or obesity, education, difficulty managing income, smoking alcohol consumption, physical activity	Being overweight or obese, having a lower education level and difficulty managing on income associated with belonging to an accumulation trajectory. Smoking, alcohol intake and physical activity level also appeared to be important risk factors for the development of some trajectories.

For peer review only - http://bmjopen.bmj.com/site/about/guidelines.xhtml

Lappenschaar et al. (2013)	Urbanization, multimorbidity at baseline	Urbanization level of a general practice is associated with the higher cumulative incidence of chronic cardiovascular conditions, in particular obesity, hypertension, dyslipidaemia, diabetes mellitus, and ischemic heart disease. Disease accumulation rate higher when multimorbidity is already present at baseline.
Perez et al. (2020)	tGSH (biomarker of multisystem failure)	Lower baseline levels of tGSH were associated with a higher rate of multimorbidity development, independent of covariates.
Quinones et al. (2011)	Race/ethnicity	White Americans differ from Black and Mexican Americans in terms of level and rate of change of multimorbidity. Mexican Americans demonstrate lower initial levels and slower accumulation of comorbidities relative to White American. In contrast, Black Americans showed an elevated level of multimorbidity throughout the 11-year period of observation, although their rate of change slowed relative to White Americans.
Quinones et al. (2019)	Race/ethnicity	Non-Hispanic black respondents had higher initial chronic disease counts, but slower accumulation rates, than non-Hispanic white respondents. Hispanic respondents had lower initial chronic disease counts but faster accumulation than non-Hispanic white respondents.
Ruel et al. (2014)	Dietary patterns	Greater amount of fruits and vegetables and grain (other than rice and wheat) associated with reduced accumulation of multimorbidity.
Ryan et al. (2018)	Multimorbidity at baseline, age, obesity, gait speed, and grip strength, access to government funded primary care	In non-multimorbid participants age, obesity, gait speed, and grip strength were significantly associated with development of multimorbidity. Age, access to government funded primary care, gait speed, and grip strength were significantly associated with worsening of multimorbidity in those with multimorbidity. Gait speed and age were significantly associated with new condition development in people with complex multimorbidity.<br>-> (Overall) Gait speed, grip strength, and age were significantly associated with both the development of multimorbidity and accrual of additional conditions with evidence of a dose-response relationship.
Siriwardhana et al. (2018)	Age, sex, race/ethnicity	Men were more likely to transition between states than women. Whites had the highest risk of transitioning from ischemic heart disease to death. Asians and Native Hawaiian and Pacific Islanders were more likely to transition from diabetes to diabetes and chronic kidney disease.
Strauss et al. (2014)	Age, deprivation	Younger age groups were more likely to be in the non-chronic cluster than older groups. Females were more likely to develop or start with multimorbidity than males. More deprived individuals were more likely to be in the evolving (rather than static) multimorbidity cluster.
Xu et al. (2018)	Sex, age, marital status, income, education, obesity, physical activity, smoking, and immigrant status.	Odds of multimorbidity progression increased over time and with age. Women with stroke were more likely to progress to another disease and become multimorbid than other baseline characteristics. In adjusted models, accumulation of MM was associated with non-married status, low income, lower education, obesity, sedentary and smoking, immigrant status. Obesity differently associated with different sequences.

For peer review only - http://bmjopen.bmj.com/site/about/guidelines.xhtml

For peer review only

Health and health behaviours also showed associations. A Chinese study showed that a greater consumption of fruits, vegetables and grain slowed the development of multimorbidity [57]. Alcohol consumption, smoking, and physical inactivity was associated with worse multimorbidity trajectory patterns [30,42,47]. Physical function (measured by gait speed and grip strength at baseline) was associated with development and worsening of multimorbidity over 2 years in a sample of adults aged 50 and over [29]. Being overweight or obese was also associated with developing or worsening multimorbidity trajectory [29,42,47]. Two studies investigated the role of specific biomarkers, finding that chronic inflammation, system dysregulation, and multisystem failure, are associated with faster rate of multimorbidity accumulation [27,34]. There were associations with family factors: being married was found to be protective of greater multimorbidity accumulation [37,54], and young parenthood (younger than 25) and extremely high parity (nine of more births) significant risk factors [46]. Finally, a negative attitude towards life and health such as low life satisfaction and negative health outlook was associated with poorer multimorbidity trajectories [32].

DISCUSSION

Understanding longitudinal multimorbidity trajectories is an important public health priority for clinicians, academics, and funders alike [4]. This review aimed to take a systematic approach to scope existing research in the field with a focus on summarizing commonly used methodological approaches and substantive findings. In doing so we provide, to our knowledge, the first review to address longitudinal studies of multimorbidity, in a field saturated by cross-sectional research [2,12,15,58]. A strength of this review is the systematic and robust approach taken to searching and screening articles for inclusion, and reviewing the selected studies, which should limit selection and extraction bias. We used pre-defined search terms, inclusion criteria, and data extraction tools, and we engaged in double screening and extraction [22]. The scoping review process meant that we summarized a wide variety of evidence, and therefore it was not possible to perform a meta-analysis or use a standardized critical appraisal tool. Nevertheless, we provide a narrative-style critical summary of the selected articles. The results demonstrate that despite widespread expressed interest, relatively few studies do take a longitudinal approach to multimorbidity. All the studies included were published within the last decade and the vast majority using data from high-income countries. The studies showed a great variability in sampling strategy, ways of measuring multimorbidity, and statistical approaches to characterizing multimorbidity longitudinally. Methods for identifying longitudinal patterns ranged from counts of diseases to cluster or group -based analyses, to modeling transitions between diseases or disease sequences, and these were differentially useful for modelling accumulation, sequencing, clusters, or transitions. From a

For peer review only - http://bmjopen.bmj.com/site/about/guidelines.xhtml

substantive perspective, the studies showed associations with adverse outcomes such as worse reported health, greater risk of disability, and mortality that we might expect based on the existing cross-sectional research. A range of multimorbidity trajectory risk factors were also identified, including socio-demographic factors, health behaviours, physical function, biomarkers, marriage and fertility factors, and attitudinal factors.

A limitation of narrative reviews is that they might select evidence to support a particular stance and do not necessarily take enough steps to eliminate selection bias. However, we selected a comprehensive set of items to extract before starting the review and we engaged in double screening and extraction. Therefore, our methodological approach should limit selection and extraction bias. In addition, our review didn't engage in a critical appraisal of the quality of the selected studies. However, the aim of the review was to scope the published evidence on longitudinal approaches to multimorbidity. Indeed, when the aim of a scoping review is to provide an overview of evidence, methodological limitations and risk of bias of the evidence are not necessarily relevant and generally not performed [18].

The review has highlighted some geographical bias in the distribution of multimorbidity research. In particular there was an underrepresentation of longitudinal multimorbidity research in low- and middle-income countries (LMICs), which likely reflects the geographical focus of cross-sectional multimorbidity research generally [59]. This may be due to underinvestment in multimorbidity research in LMICs, coupled with challenges of collecting or accessing relevant data. For example, most of selected studies use of electronic medical records or large-scale longitudinal surveys, which are rare in developing countries. Nevertheless, due to the population aging trends in LMICs, multimorbidity is already a major public health issue, with potentially more complex comorbidity patterns (e.g. undiagnosed conditions, or interactions with infectious disease), which deserve research using a longitudinal approach. Recently published work in LMICs countries has tended to employ a cross-sectional design to analyse multimorbidity [60–63], and therefore were not eligible for inclusion in this review. In addition, none of the studies in the review made cross-country comparisons, which may help to generate stronger evidence about disease trajectories and mechanisms involved in multimorbidity development and progression. For example, comparable cross-country patterns may suggest common biological mechanisms, whereas divergent findings could suggest moderation or prevention of disease processes by policy approaches to treatment, health care settings, and institutional structures.

The selected studies used a great variety of data sources including administrative data (primary and secondary care data, health insurance claim data, patient and disease registries) and survey data,

For peer review only - http://bmjopen.bmj.com/site/about/guidelines.xhtml

leading to variations in sample size and issues of generalisability. Issues of small sample size were only discussed in a limited number of studies, mostly in relation to subgroups such as ethnic minorities [23,41,49,50]. Despite the use of large surveys or administrative data, the majority of studies expressed doubts about the generalisability of their findings. For example, well-educated and wealthy individuals were reported as over-represented in longitudinal survey samples [27,29,31–33,42,47]. Studies using administrative data sources typically investigated multimorbidity based on complete follow-up and excluding those who died, generating immortal time bias and investigating potentially healthier populations [39,53]. In other studies, the choice of data sources themselves induced bias, for example where samples were based on health service users [35,48]. Others explained their sample might be representative but only of a particular group in a specific region (e.g. Utah[46]). Another issue of generalisability, mentioned in previous reviews [15], was related to the heterogenous multimorbidity measures used [64]. A wide variety of different diseases were included and only a few studies used 'standard' measurement of multimorbidity like the Charlson [20] or Elixhauser [21] indices. Due to the diversity of data sources, diseases were ascertained in multiple ways, using clinical-diagnosis, laboratory results, medication use, and self-report. The only common measurement feature was that studies in this review tended not to define multimorbidity as the presence or two or more diseases.

The choice of statistical methods served to highlight or obscure different aspects of multimorbidity. For example, the most common approach, multi- or single-level regression modelling, emphasizes accumulation, providing the opportunity to simultaneously evaluate the baseline level of multimorbidity and the (slope) change in multimorbidity, and how this differs between groups with different characteristics. However, it tends to obscure the role of specific diseases by collapsing all morbidity in a single count or index, and we cannot tell, for example, whether this faster accumulation is predominantly occurring among certain types of disorders. Complementary to regression approaches, grouped-based methodologies aimed to classify individuals into types of multimorbidity accumulation. A minority of studies employed the cluster-based approach to understand how specific diseases co-occur over time [48,54], which extends cross-sectional approaches often referred to as associative multimorbidity [11]. This has the advantage of providing a more detailed understanding of the constellation of diseases that contribute to distinct trajectories, but due to the rarity of some diseases, will tend to find only highly prevalent clusters, and is not suitable for rarer disease trajectories.

For peer review only - http://bmjopen.bmj.com/site/about/guidelines.xhtml

Some studies conceptualised longitudinal multimorbidity as transitioning between different disease states, utilizing either structured Markov frameworks [44], multistate modelling [26,41] or a more data-driven, unsupervised approaches [24,38]. The former, more structured approach to disease transition tended to provide a very detailed understanding of interactions between a small set of diseases, which can provide useful evidence for targeting prevention at those with the first disease, a risk stratification approach. The latter, data-driven approaches provide very comprehensive evidence for population-based strategies, but relies on large datasets collected over a number of years, and appropriate clinical expertise to interpret the results of patterns identified through artificial intelligence (AI). Given the growing interdisciplinary collaborations between epidemiology and computer science, data-driven research will continue to expand in the coming years and extend to prediction modelling and projections. One of the strengths of computer science, and the recent new developments in AI with machine learning, is the ability to work towards solutions that can combine prediction models and compare different treatment options for cohorts of patients (e.g. what is the likelihood that a medication commonly used for one chronic condition may speed up the progression of another condition or lead to the development of a new condition).

Compared with cross-sectional studies, longitudinal approaches provide more detailed insight about the role of specific risk factors. For example, while age is a known risk factor, this review highlights how older individuals, once multimorbid, show acceleration of multimorbidity [29]. Multimorbidity trajectory patterns varied by ethnicity [23,41,49,50], marital status [37,42], educational level, and area-level deprivation [28,33,42,47], confirming some patterns observed in cross-sectional data. A useful exploitation of longitudinal data – not included in these studies - would be to explore how change in risk factors such as SES or marital status influences different multimorbidity trajectories, which may help identify at-risk groups and target prevention strategies. As highlighted by Zhu and colleagues [55], the earlier the multimorbidity onset in the life course, the greater the life year lost for that individual. Therefore, future research should seek to take a life course approach in order to disentangle early preventable factors of multimorbidity onset but also to determine later life factors influencing additional disease accumulation. Risk factors should be considered at the level of the individual (life course and contemporaneous factors), medication use, and the wider social environment, including poor environmental conditions, and interaction with institutional structures (e.g. health care system organization). The increasing availability of 'big data' which links longitudinal administrative data on individuals with health and geospatial data will make these holistic approaches technically possible. Future research should focus on generating the knowledge required to develop interventions aimed at preventing both the onset and the worsening of multimorbidity.

For peer review only - http://bmjopen.bmj.com/site/about/guidelines.xhtml

CONCLUSION

This review identifies a small but developing body of literature attempting to describe multimorbidity longitudinally. There was a notable lack of studies in low and middle-income countries, as well exploring minority ethnic groups. A wide variety of complementary methods are employed, emphasizing factors associated with greater disease accumulation, speed of accumulation, and specific disease transition processes. Methodologies based on disease ordering or sequence was seldom explored by the studies, and while it is challenging to identify exact timing of disease, future research could seek to investigate disease sequencing that underlies the accumulation process. Risk factors for trajectory types could inform future intervention and prevention strategies at critical life course periods and disease progression turning points. Initiatives to enable researchers greater access to relevant data sources, such as the HDR UK initiative to harmonise datasets for multimorbidity research, is crucial and should become more generalised in order to gain the insight on multimorbidity processes required to feed into prevention and policy makers strategies at a global scale.

Fig 1 Caption:

Figure 1. Study selection process

For peer review only

DATA AVAILABILITY

No additional data available

AUTHOR CONTRIBUTIONS

GC participated in the screening, data extraction, and analysis and helped draft the manuscript. CM participated in the screening, data extraction, and analysis and helped draft the manuscript. FS and JB helped draft the manuscript. KK acquired the funding, conceptualised the idea for the study, participated in the screening, data extraction, and analysis and helped draft the manuscript . All authors approved the final version for submission.

ACKNOWLEDGEMENTS

We thank Iris Ho and Bruce Guthrie for providing additional references that our systematic search might have missed.

FUNDING

This work was supported by the Academy of Medical Sciences (AMS), the Wellcome Trust, the Government Department of Business, Energy and Industrial Strategy (BEIS), the British Heart Foundation Diabetes UK, and the Global Challenges Research Fund (GCRF) [Grant number SBF004\1093 awarded to Katherine Keenan]. The funders had no input to the design or execution of the study.

DECLARATIONS OF INTEREST

None.

For peer review only

REFERENCES

1 Diederichs C, Berger K, Bartels DB. The Measurement of Multiple Chronic Diseases--A Systematic Review on Existing Multimorbidity Indices. *Journals Gerontol Ser A Biol Sci Med Sci* 2011;**66A**:301–11. doi:10.1093/gerona/glq208

2 Johnston MC, Crilly M, Black C, *et al.* Defining and measuring multimorbidity: a systematic review of systematic reviews. *Eur J Public Health* 2019;**29**:182–9. doi:10.1093/eurpub/cky098

3 Hajat C, Stein E. The global burden of multiple chronic conditions: A narrative review. Prev. Med. Reports. 2018;**12**:284–93. doi:10.1016/j.pmedr.2018.10.008

4 Academy of Medical Sciences. Multimorbidity: A Priority for Global Health Research. London: 2018. https://acmedsci.ac.uk/file-download/82222577

5 Kingston A, Robinson L, Booth H, *et al.* Projections of multi-morbidity in the older population in England to 2035: estimates from the Population Ageing and Care Simulation (PACSim) model. *Age Ageing* 2018;**47**:374–80. doi:10.1093/ageing/afx201

6 Makovski TT, Schmitz S, Zeegers MP, *et al.* Multimorbidity and quality of life: Systematic literature review and meta-analysis. Ageing Res. Rev. 2019;**53**:100903. doi:10.1016/j.arr.2019.04.005

7 Ryan A, Wallace E, O'Hara P, *et al.* Multimorbidity and functional decline in community-dwelling adults: A systematic review. *Health Qual Life Outcomes* 2015;**13**:1–13. doi:10.1186/s12955-015-0355-9

8 Nunes BP, Flores TR, Mielke GI, *et al.* Multimorbidity and mortality in older adults: A systematic review and meta-analysis. Arch. Gerontol. Geriatr. 2016;**67**:130–8. doi:10.1016/j.archger.2016.07.008

9 Wang L, Si L, Cocker F, *et al.* A Systematic Review of Cost-of-Illness Studies of Multimorbidity. Appl. Health Econ. Health Policy. 2018;**16**:15–29. doi:10.1007/s40258-017-0346-6

10 Ng SK, Tawiah R, Sawyer M, *et al.* Patterns of multimorbid health conditions: a systematic review of analytical methods and comparison analysis. *Int J Epidemiol* 2018;**47**:1687–704. doi:10.1093/ije/dyy134

11 Prados-Torres A, Calderón-Larrañaga A, Hancco-Saavedra J, *et al.* Multimorbidity patterns: a systematic review. *J Clin Epidemiol* 2014;**67**:254–66. doi:10.1016/J.JCLINEPI.2013.09.021

12 Violan C, Foguet-Boreu Q, Flores-Mateo G, *et al.* Prevalence, Determinants and Patterns of Multimorbidity in Primary Care: A Systematic Review of Observational Studies. *PLoS One* 2014;**9**:e102149. doi:10.1371/journal.pone.0102149

13 Pathirana TI, Jackson CA. Socioeconomic status and multimorbidity: a systematic review and meta-analysis. *Aust N Z J Public Health* 2018;**42**:186–94. doi:10.1111/1753-6405.12762

For peer review only - http://bmjopen.bmj.com/site/about/guidelines.xhtml

14 Fortin M, Haggerty J, Almirall J, *et al.* Lifestyle factors and multimorbidity: A cross sectional study. *BMC Public Health* 2014;**14**:1–8. doi:10.1186/1471-2458-14-686

15 Xu X, Mishra GD, Jones M. Mapping the global research landscape and knowledge gaps on multimorbidity: a bibliometric study. *J Glob Health* 2017;**7**:010414. doi:10.7189/jogh.07.010414

16 Moher D, Stewart L, Shekelle P. All in the Family: Systematic reviews, rapid reviews, scoping reviews, realist reviews, and more. Syst. Rev. 2015;**4**:183. doi:10.1186/s13643-015-0163-7

17 Munn Z, Peters MDJ, Stern C, *et al.* Systematic review or scoping review? Guidance for authors when choosing between a systematic or scoping review approach. *BMC Med Res Methodol* 2018;**18**:1–7. doi:10.1186/s12874-018-0611-x

18 Peters MDJ, Godfrey CM, Khalil H, *et al.* Guidance for conducting systematic scoping reviews. *Int J Evid Based Healthc* 2015;**13**:141–6. doi:10.1097/XEB.0000000000000050

19 Tricco AC, Lillie E, Zarin W, *et al.* PRISMA Extension for Scoping Reviews (PRISMA-ScR): Checklist and Explanation. *Ann Intern Med* 2018;**169**:467. doi:10.7326/M18-0850

20 Charlson ME, Pompei P, Ales KL, *et al.* A new method of classifying prognostic comorbidity in longitudinal studies: Development and validation. *J Chronic Dis* 1987;**40**:373–83. doi:10.1016/0021-9681(87)90171-8

21 Elixhauser A, Steiner C, Harris DR, *et al.* Comorbidity Measures for Use with Administrative Data. *Med Care* 1998;**36**:8–27. doi:10.1097/00005650-199801000-00004

22 Buscemi N, Hartling L, Vandermeer B, *et al.* Single data extraction generated more errors than double data extraction in systematic reviews. *J Clin Epidemiol* 2006;**59**:697–703. doi:10.1016/j.jclinepi.2005.11.010

23 Ashworth M, Durbaba S, Whitney D, *et al.* Journey to multimorbidity: Longitudinal analysis exploring cardiovascular risk factors and sociodemographic determinants in an urban setting. *BMJ Open* 2019;**9**:e031649. doi:10.1136/bmjopen-2019-031649

24 Beck MK, Jensen AB, Nielsen AB, *et al.* Diagnosis trajectories of prior multi-morbidity predict sepsis mortality. *Sci Rep* 2016;**6**:1–9. doi:10.1038/srep36624

25 Lappenschaar M, Hommersom A, Lucas PJF, *et al.* Multilevel temporal Bayesian networks can model longitudinal change in multimorbidity. *J Clin Epidemiol* 2013;**66**:1405–16. doi:10.1016/j.jclinepi.2013.06.018

26 Lindhagen L, Van Hemelrijck M, Robinson D, *et al.* How to model temporal changes in comorbidity for cancer patients using prospective cohort data. *BMC Med Inform Decis Mak* 2015;**15**:96. doi:10.1186/s12911-015-0217-8

27 Pérez LM, Hooshmand B, Mangialasche F, *et al.* Glutathione Serum Levels and Rate of

For peer review only

Multimorbidity Development in Older Adults. *Journals Gerontol Ser A* 2020;**75**:1089–94. doi:10.1093/gerona/glz101

28 Strauss VY, Jones PW, Kadam UT, *et al.* Distinct trajectories of multimorbidity in primary care were identified using latent class growth analysis. *J Clin Epidemiol* 2014;**67**:1163–71. doi:10.1016/j.jclinepi.2014.06.003

29 Ryan A, Murphy C, Boland F, *et al.* What Is the Impact of Physical Activity and Physical Function on the Development of Multimorbidity in Older Adults Over Time? A Population-Based Cohort Study. *Journals Gerontol Ser A* 2018;**73**:1538–44. doi:10.1093/gerona/glx251

30 Freisling H, Viallon V, Lennon H, *et al.* Lifestyle factors and risk of multimorbidity of cancer and cardiometabolic diseases: A multinational cohort study. *BMC Med* 2020;**18**:1–11. doi:10.1186/s12916-019-1474-7

31 Calderón-Larrañaga A, Santoni G, Wang HX, *et al.* Rapidly developing multimorbidity and disability in older adults: does social background matter? *J Intern Med* 2018;**283**:489–99. doi:10.1111/joim.12739

32 Calderón-Larrañaga A, Vetrano DL, Welmer AK, *et al.* Psychological correlates of multimorbidity and disability accumulation in older adults. *Age Ageing* 2019;**48**:789–96. doi:10.1093/ageing/afz117

33 Dekhtyar S, Vetrano DL, Marengoni A, *et al.* Association Between Speed of Multimorbidity Accumulation in Old Age and Life Experiences: A Cohort Study. *Am J Epidemiol* 2019;**188**:1627–36. doi:10.1093/aje/kwz101

34 Fabbri E, An Y, Zoli M, *et al.* Aging and the Burden of Multimorbidity: Associations With Inflammatory and Anabolic Hormonal Biomarkers. *Journals Gerontol Ser A* 2015;**70**:63–70. doi:10.1093/gerona/glu127

35 Fraccaro P, Kontopantelis E, Sperrin M, *et al.* Predicting mortality from change-over-time in the Charlson Comorbidity Index: A retrospective cohort study in a data-intensive UK health system. *Med (United States)* 2016;**95**. doi:10.1097/MD.0000000000004973

36 Gellert P, Von Berenberg P, Oedekoven M, *et al.* Centenarians Differ in Their Comorbidity Trends during the 6 Years before Death Compared to Individuals Who Died in Their 80s or 90s. *Journals Gerontol - Ser A Biol Sci Med Sci* 2018;**73**:1357–62. doi:10.1093/gerona/glx136

37 Hiyoshi A, Fall K, Bergh C, *et al.* Comorbidity trajectories in working age cancer survivors: A national study of Swedish men. *Cancer Epidemiol* 2017;**48**:48–55. doi:10.1016/j.canep.2017.03.001

38 Jensen AB, Moseley PL, Oprea TI, *et al.* Temporal disease trajectories condensed from population-wide registry data covering 6.2 million patients. *Nat Commun* 2014;**5**.

For peer review only

For peer review only - http://bmjopen.bmj.com/site/about/guidelines.xhtml

doi:10.1038/ncomms5022

39 Faruqui SHA, Alaeddini A, Jaramillo CA, *et al.* Mining patterns of comorbidity evolution in patients with multiple chronic conditions using unsupervised multi-level temporal Bayesian network. *PLoS One* 2018;**13**:e0199768. doi:10.1371/journal.pone.0199768

40 Canizares M, Hogg-Johnson S, Gignac MAM, *et al.* Increasing trajectories of multimorbidity over time: Birth cohort differences and the role of changes in obesity and income. *Journals Gerontol - Ser B Psychol Sci Soc Sci* 2018;**73**:1303–14. doi:10.1093/geronb/gbx004

41 Siriwardhana C, Lim E, Davis J, *et al.* Progression of diabetes, ischemic heart disease, and chronic kidney disease in a three chronic conditions multistate model. *BMC Public Health* 2018;**18**:752. doi:10.1186/s12889-018-5688-y

42 Xu X, Mishra GD, Dobson AJ, *et al.* Progression of diabetes, heart disease, and stroke multimorbidity in middle-aged women: A 20-year cohort study. *PLoS Med* 2018;**15**:e1002516. doi:10.1371/journal.pmed.1002516

43 Zeng C, Ellis JL, Steiner JF, *et al.* Assessment of morbidity over time in predicting health outcomes. *Med Care* 2014;**52**. doi:10.1097/MLR.0000000000000033

44 Alaeddini A, Jaramillo CA, Faruqui SHA, *et al.* Mining major transitions of chronic conditions in patients with multiple chronic conditions. *Methods Inf Med* 2017;**56**:391–400. doi:10.3414/ME16-01-0135

45 Fabbri E, An Y, Zoli M, *et al.* Association Between Accelerated Multimorbidity and Age-Related Cognitive Decline in Older Baltimore Longitudinal Study of Aging Participants without Dementia. *J Am Geriatr Soc* 2016;**64**:965–72. doi:10.1111/jgs.14092

46 Hanson HA, Smith KR, Zimmer Z. Reproductive History and Later-Life Comorbidity Trajectories: A Medicare-Linked Cohort Study From the Utah Population Database. *Demography* 2015;**52**:2021–49. doi:10.1007/s13524-015-0439-5

47 Jackson CA, Dobson A, Tooth L, *et al.* Body mass index and socioeconomic position are associated with 9-year trajectories of multimorbidity: A population-based study. *Prev Med (Baltim)* 2015;**81**:92–8. doi:10.1016/j.ypmed.2015.08.013

48 Pugh MJ, Finley EP, Wang CP, *et al.* A retrospective cohort study of comorbidity trajectories associated with traumatic brain injury in veterans of the Iraq and Afghanistan wars. *Brain Inj* 2016;**30**:1481–90. doi:10.1080/02699052.2016.1219055

49 Quiñones AR, Botoseneanu A, Markwardt S, *et al.* Racial/ethnic differences in multimorbidity development and chronic disease accumulation for middle-aged adults. *PLoS One* 2019;**14**:e0218462. doi:10.1371/journal.pone.0218462

50 Quinones AR, Liang J, Bennett JM, *et al.* How Does the Trajectory of Multimorbidity Vary

For peer review only

Across Black, White, and Mexican Americans in Middle and Old Age? *Journals Gerontol Ser B Psychol Sci Soc Sci* 2011;**66B**:739–49. doi:10.1093/geronb/gbr106

51 Rocca WA, Gazzuola-Rocca L, Smith CY, *et al.* Accelerated Accumulation of Multimorbidity After Bilateral Oophorectomy: A Population-Based Cohort Study. *Mayo Clin Proc* 2016;**91**:1577–89. doi:10.1016/j.mayocp.2016.08.002

52 Ruel G, Lévesque J-F, Stocks N, *et al.* Understanding the Evolution of Multimorbidity: Evidences from the North West Adelaide Health Longitudinal Study (NWAHS). *PLoS One* 2014;**9**:e96291. doi:10.1371/journal.pone.0096291

53 Chang HY, Clark JM, Weiner JP. Morbidity trajectories as predictors of utilization: Multi-year disease patterns in taiwan's national health insurance program. *Med Care* 2011;**49**:918–23. doi:10.1097/MLR.0b013e31821b344f

54 Hsu HC. Trajectories of multimorbidity and impacts on successful aging. *Exp Gerontol* 2015;**66**:32–8. doi:10.1016/j.exger.2015.04.005

55 Zhu Z, Heng BH, Teow KL. Lifetime trajectory simulation of chronic disease progression and comorbidity development. *J Biomed Inform* 2018;**88**:29–36. doi:10.1016/j.jbi.2018.11.002

56 Kim JH, Rhee Y, Lee Y. Longitudinal changes in comorbidity patterns over time in relation to mortality in older Korean adults. *Arch Gerontol Geriatr* 2018;**79**:63–8. doi:10.1016/j.archger.2018.08.004

57 Ruel G, Shi Z, Zhen S, *et al.* Association between nutrition and the evolution of multimorbidity: The importance of fruits and vegetables and whole grain products. *Clin Nutr* 2014;**33**:513–20. doi:10.1016/j.clnu.2013.07.009

58 Nguyen H, Manolova G, Daskalopoulou C, *et al.* Prevalence of multimorbidity in community settings: A systematic review and meta-analysis of observational studies. *J Comorbidity* 2019;**9**:2235042X1987093. doi:10.1177/2235042x19870934

59 Abebe F, Schneider M, Asrat B, *et al.* Multimorbidity of chronic non-communicable diseases in low- and middle-income countries: A scoping review. *J Comorbidity* 2020;**10**:2235042X2096191. doi:10.1177/2235042x20961919

60 Bayes-Marin I, Sanchez-Niubo A, Egea-Cortés L, *et al.* Multimorbidity patterns in low-middle and high income regions: A multiregion latent class analysis using ATHLOS harmonised cohorts. *BMJ Open* 2020;**10**:34441. doi:10.1136/bmjopen-2019-034441

61 Garin N, Koyanagi A, Chatterji S, *et al.* Global Multimorbidity Patterns: A Cross-Sectional, Population-Based, Multi-Country Study. *Journals Gerontol Ser A Biol Sci Med Sci* 2016;**71**:205–14. doi:10.1093/gerona/glv128

62 Pati S, Swain S, Metsemakers J, *et al.* Pattern and severity of multimorbidity among patients

For peer review only - http://bmjopen.bmj.com/site/about/guidelines.xhtml

attending primary care settings in Odisha, India. *PLoS One* 2017;**12**:e0183966. doi:10.1371/journal.pone.0183966

63 Rivera-Almaraz A, Manrique-Espinoza B, Ávila-Funes JA, *et al.* Disability, quality of life and all-cause mortality in older Mexican adults: Association with multimorbidity and frailty. *BMC Geriatr* 2018;**18**:1–9. doi:10.1186/s12877-018-0928-7

64 Stirland LE, González-Saavedra L, Mullin DS, *et al.* Measuring multimorbidity beyond counting diseases: Systematic review of community and population studies and guide to index choice. *BMJ* 2020;**368**. doi:10.1136/bmj.m160

For peer review only

For peer review only - http://bmjopen.bmj.com/site/about/guidelines.xhtml

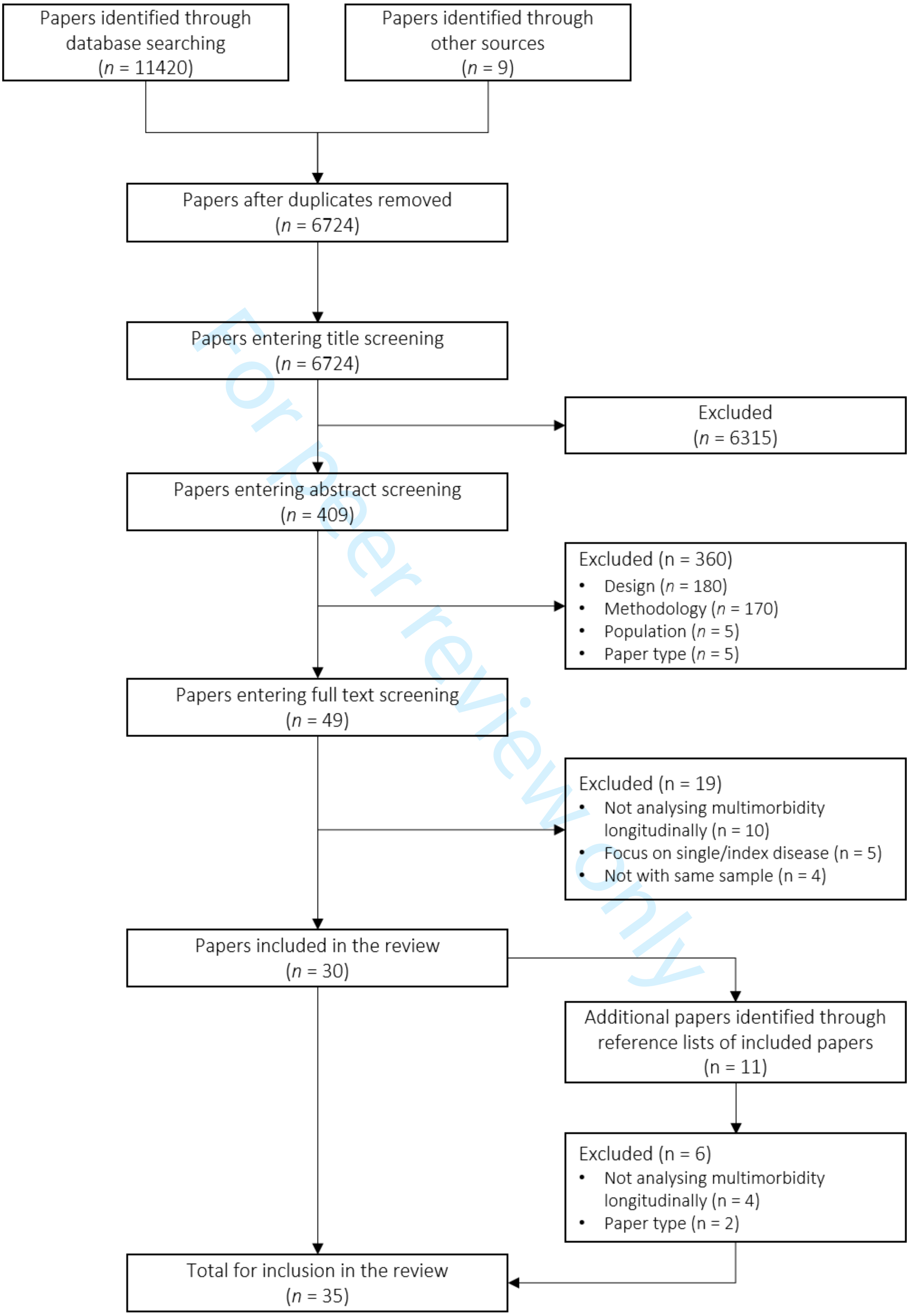

For peer review only

Appendix A: Preferred Reporting Items for Systematic reviews and Meta-Analyses extension for Scoping Reviews (PRISMA-ScR) Checklist

SECTION	ITEM	PRISMA-ScR CHECKLIST ITEM	REPORTED ON PAGE #
**TITLE**			
Title	1	Identify the report as a scoping review.	1
**ABSTRACT**			
Structured summary	2	Provide a structured summary that includes (as applicable): background, objectives, eligibility criteria, sources of evidence, charting methods, results, and conclusions that relate to the review questions and objectives.	2
**INTRODUCTION**			
Rationale	3	Describe the rationale for the review in the context of what is already known. Explain why the review questions/objectives lend themselves to a scoping review approach.	4-5
Objectives	4	Provide an explicit statement of the questions and objectives being addressed with reference to their key elements (e.g., population or participants, concepts, and context) or other relevant key elements used to conceptualize the review questions and/or objectives.	-5
**METHODS**			
Protocol and registration	5	Indicate whether a review protocol exists; state if and where it can be accessed (e.g., a Web address); and if available, provide registration information, including the registration number.	n/a
Eligibility criteria	6	Specify characteristics of the sources of evidence used as eligibility criteria (e.g., years considered, language, and publication status), and provide a rationale.	5
Information sources*	7	Describe all information sources in the search (e.g., databases with dates of coverage and contact with authors to identify additional sources), as well as the date the most recent search was executed.	7 (May 2020)
Search	8	Present the full electronic search strategy for at least 1 database, including any limits used, such that it could be repeated.	Table 2 and Appendix B
Selection of sources of evidence†	9	State the process for selecting sources of evidence (i.e., screening and eligibility) included in the scoping review.	5-8
Data charting process‡	10	Describe the methods of charting data from the included sources of evidence (e.g., calibrated forms or forms that have been tested by the team before their use, and whether data charting was done independently or in duplicate) and any processes for obtaining and confirming data from investigators.	7
Data items	11	List and define all variables for which data were sought and any assumptions and simplifications made.	7-8
Critical appraisal of individual sources of evidence§	12	If done, provide a rationale for conducting a critical appraisal of included sources of evidence; describe the methods used and how this information was used in any data synthesis (if appropriate).	n/a
Synthesis of results	13	Describe the methods of handling and summarizing the data that were charted.	7-8

For peer review only - http://bmjopen.bmj.com/site/about/guidelines.xhtml

SECTION	ITEM	PRISMA-ScR CHECKLIST ITEM	REPORTED ON PAGE #
**RESULTS**			
Selection of sources of evidence	14	Give numbers of sources of evidence screened, assessed for eligibility, and included in the review, with reasons for exclusions at each stage, ideally using a flow diagram.	8 and Figure 1
Characteristics of sources of evidence	15	For each source of evidence, present characteristics for which data were charted and provide the citations.	9 and Table 3
Critical appraisal within sources of evidence	16	If done, present data on critical appraisal of included sources of evidence (see item 12).	n/a
Results of individual sources of evidence	17	For each included source of evidence, present the relevant data that were charted that relate to the review questions and objectives.	Tables 4, 5, 7
Synthesis of results	18	Summarize and/or present the charting results as they relate to the review questions and objectives.	12-23
**DISCUSSION**			
Summary of evidence	19	Summarize the main results (including an overview of concepts, themes, and types of evidence available), link to the review questions and objectives, and consider the relevance to key groups.	23-24
Limitations	20	Discuss the limitations of the scoping review process.	24
Conclusions	21	Provide a general interpretation of the results with respect to the review questions and objectives, as well as potential implications and/or next steps.	24-27
**FUNDING**			
Funding	22	Describe sources of funding for the included sources of evidence, as well as sources of funding for the scoping review. Describe the role of the funders of the scoping review.	28

JBI = Joanna Briggs Institute; PRISMA-ScR = Preferred Reporting Items for Systematic reviews and Meta-Analyses extension for Scoping Reviews.
* Where *sources of evidence* (see second footnote) are compiled from, such as bibliographic databases, social media platforms, and Web sites.
† A more inclusive/heterogeneous term used to account for the different types of evidence or data sources (e.g., quantitative and/or qualitative research, expert opinion, and policy documents) that may be eligible in a scoping review as opposed to only studies. This is not to be confused with *information sources* (see first footnote).
‡ The frameworks by Arksey and O'Malley (6) and Levac and colleagues (7) and the JBI guidance (4, 5) refer to the process of data extraction in a scoping review as data charting.
§ The process of systematically examining research evidence to assess its validity, results, and relevance before using it to inform a decision. This term is used for items 12 and 19 instead of "risk of bias" (which is more applicable to systematic reviews of interventions) to include and acknowledge the various sources of evidence that may be used in a scoping review (e.g., quantitative and/or qualitative research, expert opinion, and policy document).

From: Tricco AC, Lillie E, Zarin W, O'Brien KK, Colquhoun H, Levac D, et al. PRISMA Extension for Scoping Reviews (PRISMAScR): Checklist and Explanation. Ann Intern Med. 2018;169:467–473. doi: 10.7326/M18-0850.

For peer review only - http://bmjopen.bmj.com/site/about/guidelines.xhtml

Appendix B: Data Search Strategies

Medline

1. exp comorbidity/
2. exp multiple chronic conditions/
3. (co-morbid* or comorbid* or multi-morbid* or multimorbid or co-occur* or cooccur*).mp.
4. 1 or 2 or 3
5. exp cohort studies/
6. 4 and 5
7. ((disease or condition or illness) and (cluster* or trajector* or cascade* or accumulat* or combination* or sequenc* or transition*)).mp.
8. 6 and 7
9. limit 8 to (english language and humans and ("adult (19 to 44 years)" or "young adult and adult (19-24 and 19-44)" or "middle age (45 to 64 years)" or "middle aged (45 plus years)" or "all aged (65 and over)" or "aged (80 and over)"))
10. 9 not (dna or cell* or gene or genes or bater* or covid*).mp.

Web of Science

1. TOPIC: (co-morbid* or comorbid* or multi-morbid* or multimorbid* or co-occur* or cooccur*)
2. TOPIC: (cohort* or longitudinal* or prospective*)
3. TOPIC: ((disease or condition or illness) and (cluster* or trajector* or cascade* or accumulat* or combination* or sequenc* or transition*))
4. 1 and 2
5. 3 and 4
6. 5 Refined by: Web of Science Index: (WOS.SCI or WOS.SSCI) and Languages: (English) and Document type: (Article)
7. Topic: (cell* or gene or genes or bacter* or DNA or covid*)
8. 6 not 7

Embase

1. (co-morbid* or comorbid* or multi-morbid* or multimorbid or co-occur* or cooccur*).mp.
2. exp multiple chronic conditions/
3. 1 or 2
4. prospective study/
5. cohort study/
6. exp longitudinal study/
7. 4 or 5 or 6
8. 3 and 7
9. ((disease or condition or illness) and (cluster* or trajector* or cascade* or accumulat* or combination* or sequenc* or transition*)).mp.
10. 8 and 9
11. limit 10 to (human and english language and (article or article in press) and (adult <18 to 64 years> or aged <65+ years>))
12. 11 not (dna or cell* or bacter* or gene or genes or covid*).mp.

Scopus

For peer review only

((TITLE-ABS-KEY (co-morbid* OR comorbid* OR multi-morbid* OR multimorbid* OR co-occur* OR cooccur*)) AND (TITLE-ABS-KEY ((disease OR condition OR illness) AND (cluster* OR trajector* OR cascade * OR accumulat* OR combination* OR sequenc* OR transition*))) AND (TITLE-ABS-KEY (cohort* OR longitudinal* OR prospective*))) AND NOT (TITLE-ABS KEY (cell* OR gene OR genes OR bacter* OR dna OR covid*)) AND (LIMIT-TO (DOCTYPE , "ar")) AND (LIMIT-TO (SUBJAREA , "MEDI") OR LIMIT-TO (SUBJAREA , "PSYC") OR LIMIT-TO (SUBJAREA , "NURS") OR LIMIT-TO (SUBJAREA , "MULT") OR LIMIT-TO (SUBJAREA , "HEAL") OR LIMIT-TO (SUBJAREA , "SOCI") OR LIMIT-TO (SUBJAREA , "COMP") OR LIMIT-TO (SUBJAREA , "DECI")) AND (LIMIT-TO (EXACTKEYWORD , "Human") OR LIMIT-TO (EXACTKEYWORD , "Humans")) AND (LIMIT-TO (LANGUAGE , "English")) AND (LIMIT-TO (SRCTYPE , "j"))

For peer review only

For peer review only - http://bmjopen.bmj.com/site/about/guidelines.xhtml

Appendix C: List of conditions included in each selected study

Study, year	List of diseases or disease categories included if available
Alaeddini et al. (2017)	Back pain, hypertension, Post-Traumatic Stress Disorder (PTSD), and depression
Ashworth et al. (2019)	Long-term conditions: atrial fibrillation, COPD, chronic pain, Chronic Kidney Disease (CKD), Coronary Heart Disease (CHD), Diabetes Mellitus (DM), dementia, depression, Heart Failure (HF), serious mental illness, stroke, and morbid obesity
Beck et al. (2016)	All temporal trajectory of four diseases, identified from temporal directed pairs of diseases combined into longer trajectories of temporal consecutives diseases (three temporal disease pairs) with sepsis as the fourth disease, trajectory must be found for at least 20 patients following Jensen (2014) methodology
Calderon-Larranaga et al. (2018)	918 chronic conditions identified by an international team of geriatricians, general practitioners and epidemiologists. A condition was selected if defined as chronic and if it either worsened quality of life, residual disability remained or if long period of care, treatment or rehabilitation was required.
Calderon-Larranaga et al. (2019)	As specified by Calderon-Larranaga et al. (2018)
Canizares et al. (2017)	Chronic conditions: arthritis, back problems, asthma, allergies (excluding food allergies), bronchitis, emphysema, diabetes, high blood pressure, heart conditions, stroke, cancer, ulcers, urinary incontinency, dementia, migraine, glaucoma, and cataracts.
Chang et al. (2011)	27 morbidity patterns grouped into 6 trajectory groups: constant high, constant medium, constant low, decreasing, increasing, and erratic.
Dekhtyar et al. (2019)	As specified by Calderon-Larranaga et al. (2018)
Fabbri et al. (2015)	Chronic conditions: hypertension, diabetes, ischemic heart disease, congestive heart failure, stroke, COPD, cancer, Parkinson's disease, hip fracture, lower extremities joint disease, anemia, chronic kidney disease, peripheral arterial disease, cognitive impairment, and depression.
Fabbri et al. (2016)	Chronic diseases: hypertension, diabetes, ischemic heart disease, congestive heart failure, stroke, COPD, cancer, Parkinson's disease, hip fracture, lower extremities joint disease, anemia, chronic kidney disease, and peripheral arterial disease.
Faruqui et al. (2018)	Traumatic brain injury (TBI), Post-Traumatic Stress Disorder (PTSD), depression, substance abuse, and back pain.
Fraccaro et al. (2016)	A list of 22 diseases of the CCI score is used but due to data restrictions on sexual and mental health, diseases such as HIV and dementia could not be included
Freisling et al. (2020)	Cancer, CVD, Type 2 Diabetes
Gellert et al. (2018)	A list of 30 health conditions based on the Elixhauser multimorbidity index
Hanson et al. (2015)	A list of 17 conditions based on the Charlson comorbidity index: myocardial infarction, congestive heart failure, peripheral vascular disease, cerebrovascular disease, dementia, chronic pulmonary disease, rheumatologic disease, peptic ulcer disease, mild liver disease, diabetes (mild to moderate), diabetes with chronic complications, hemiplegia or paraplegia, renal kidney disease, any malignancy, moderate or severe liver disease, metastatic solid tumor, AIDS
Hiyoshi et al. (2017)	List of diseases including some based on the Charlson Comorbidity Index: depression, anxiety, osteoporosis, and infectious disease<br>+ myocardial infarction, congestive heart failure, peripheral vascular disease, cerebrovascular disease, dementia, chronic pulmonary disease, rheumatologic disease, chronic renal failure, mild liver disease, diabetes with and without chronic complications, hemiplegia or paraplegia, renal disease, moderate or severe liver disease, and AIDS/HIV
Hsu (2015)	Diseases were grouped into six disease types: CVD (including diabetes mellitus, heart disease, and stroke), chronic non-specific lung disease (CNSLD, including lung disease, asthma, bronchitis, and emphysema), gastrointestinal disease (GI, including liver disease, gallbladder disease, and gastrointestinal disorders), arthritis (or rheumatism), cancer, and renal disease.
Jackson et al. (2015)	Diabetes, impaired glucose tolerance, osteoarthritis, rheumatoid arthritis, other arthritis, heart disease, hypertension, stroke, asthma, bronchitis or emphysema, osteoporosis, breast cancer, cervical cancer, other cancer, depression, anxiety or nervous disorder, and other psychiatric condition and chronic fatigue syndrome
Jensen et al. (2014)	All diseases taken at three level ICD 10 code
Kim et al. (2018)	Metastatic cancer, congestive heart failure, dementia, renal failure, weight loss, hemiplegia, alcohol abuse, any tumour, cardiac arrhythmias, chronic pulmonary disease, coagulopathy, complicated diabetes, deficiency anaemias, fluid and electrolyte disorders, liver disease, peripheral vascular disorder, psychosis, pulmonary circulation disorders, HIV/AIDS, hypertension
Lappenschaar et al. (2013)	Diabetes mellitus, heart failure, stroke, ischemic heart disease, retinopathy, and nephropathy<br>Obesity, hypertension, and lipid disorder used as health risk

For peer review only - http://bmjopen.bmj.com/site/about/guidelines.xhtml

Lindhagen et al. (2015)	A list of 17 groups of diseases based on the Charlson Comorbidity Index
Perez et al. (2020)	As specified by Calderon-Larranaga et al. (2018)
Pugh et al. (2016)	Traumatic brain injury and a list of diseases with include "physical thyroid, obesity, hypertension, diabetes, chronic lung disease, cardiac conditions) and mental health (depression, anxiety, SUD, PTSD, bipolar disorder) conditions as well as other conditions such as hearing/tinnitus/vision disorders, headache, other pain, insomnia, memory loss, dementia, seizures, spinal cord injury/amputations/burns.
Quinones et al. (2011)	Hypertension, heart disease, diabetes, cancer, lung disease, arthritis, and stroke
Quinones et al. (2019)	Heart disease (including myocardial infarction, coronary heart disease, angina, congestive heart failure, or other heart problems), hypertension, stroke (excluding transient ischemic attack), diabetes, arthritis, lung disease (including chronic bronchitis or emphysema and excluding asthma), and cancer (including any malignant tumours with the exception of skin cancer).
Rocca et al. (2016)	Depression, anxiety, substance abuse disorders, dementia, schizophrenia, hyperlipidemia, hypertension, diabetes, cardiac arrhythmia, coronary artery disease, stroke, congestive heart failure, arthritis, cancer (all types), asthma, chronic obstructive pulmonary disease, osteoporosis, chronic kidney disease.
Ruel et al. (2014)	Asthma, cardiovascular disease & stroke (CVD), chronic obstructive pulmonary disease (COPD), diabetes, mood and anxiety disorders, any other mental disorders, hypercholesterolemia, and hypertension.
Ruel et al. (2014)	Anemia, hypertension, hypercholesterolemia, diabetes, arthritis, hepatitis, coronary heart disease, asthma, stroke, fracture, and cancer.
Ryan et al. (2018)	The list of 16 chronic conditions groups: cardiac conditions, cerebrovascular conditions, hypertension, diabetes, high cholesterol, chronic respiratory disease, liver disease, eye disease, cognitive impairment, arthritis, osteoporosis, cancer, Parkinson's disease, emotional/psychological condition, stomach ulcers, and varicose veins.
Siriwardhana et al. (2018)	Diabetes, ischemic heart disease, and chronic kidney disease
Strauss et al. (2014)	A list of 42 chronic and progressive morbidities as validated by a clinical consensus exercise with eight GPs: benign neoplasm of prostate, carcinoma in situ, neurofibromatosis, hypothyroidism, diabetes mellitus, pure hypercholesterolaemia, obesity, hereditary haemolytic anaemia, senile/presenile dementia, hereditary and idiopathic peripheral neuropathy, primary angle glaucoma, cataract, corneal opacity and disorders of cornea, deafness, mitral stenosis, rheumatic heart disease, high blood pressure, hypertensive heart disease, hypertensive renal disease, angina pectoris, ischaemic heart disease, atrial fibrillation, congestive heart failure, cerebral atherosclerosis, cerebrovascular disease, intermittent claudication, chronic bronchitis, emphysema, extrinsic allergic alveolitis, pulmonary oedema, diffuse pulmonary fibrosis, nephritis and nephropathy, hydronephrosis, prostatism, rheumatoid arthritis, arthropathy, cervical spondylosis, Paget's disease of bone, osteoporosis, Nervous system injury, war injuries
Xu et al. (2018)	Diabetes mellitus, heart disease (heart attack, angina), and stroke
Zeng et al. (2014)	The list of 17 chronic conditions of the Charlson Comorbidity Index
Zhu et al. (2018)	Diabetes type 2, hypertension, chronic kidney disease, coronary heart disease, stroke, and heart failure

For peer review only

Appendix A: Preferred Reporting Items for Systematic reviews and Meta-Analyses extension for Scoping Reviews (PRISMA-ScR) Checklist

SECTION	ITEM	PRISMA-ScR CHECKLIST ITEM	REPORTED ON PAGE #
**TITLE**			
Title	1	Identify the report as a scoping review.	1
**ABSTRACT**			
Structured summary	2	Provide a structured summary that includes (as applicable): background, objectives, eligibility criteria, sources of evidence, charting methods, results, and conclusions that relate to the review questions and objectives.	2
**INTRODUCTION**			
Rationale	3	Describe the rationale for the review in the context of what is already known. Explain why the review questions/objectives lend themselves to a scoping review approach.	4-5
Objectives	4	Provide an explicit statement of the questions and objectives being addressed with reference to their key elements (e.g., population or participants, concepts, and context) or other relevant key elements used to conceptualize the review questions and/or objectives.	-5
**METHODS**			
Protocol and registration	5	Indicate whether a review protocol exists; state if and where it can be accessed (e.g., a Web address); and if available, provide registration information, including the registration number.	n/a
Eligibility criteria	6	Specify characteristics of the sources of evidence used as eligibility criteria (e.g., years considered, language, and publication status), and provide a rationale.	5
Information sources*	7	Describe all information sources in the search (e.g., databases with dates of coverage and contact with authors to identify additional sources), as well as the date the most recent search was executed.	7 (May 2020)
Search	8	Present the full electronic search strategy for at least 1 database, including any limits used, such that it could be repeated.	Table 2 and Appendix B
Selection of sources of evidence†	9	State the process for selecting sources of evidence (i.e., screening and eligibility) included in the scoping review.	5-8
Data charting process‡	10	Describe the methods of charting data from the included sources of evidence (e.g., calibrated forms or forms that have been tested by the team before their use, and whether data charting was done independently or in duplicate) and any processes for obtaining and confirming data from investigators.	7
Data items	11	List and define all variables for which data were sought and any assumptions and simplifications made.	7-8
Critical appraisal of individual sources of evidence§	12	If done, provide a rationale for conducting a critical appraisal of included sources of evidence; describe the methods used and how this information was used in any data synthesis (if appropriate).	n/a
Synthesis of results	13	Describe the methods of handling and summarizing the data that were charted.	7-8

For peer review only

SECTION	ITEM	PRISMA-ScR CHECKLIST ITEM	REPORTED ON PAGE #
**RESULTS**			
Selection of sources of evidence	14	Give numbers of sources of evidence screened, assessed for eligibility, and included in the review, with reasons for exclusions at each stage, ideally using a flow diagram.	8 and Figure 1
Characteristics of sources of evidence	15	For each source of evidence, present characteristics for which data were charted and provide the citations.	9 and Table 3
Critical appraisal within sources of evidence	16	If done, present data on critical appraisal of included sources of evidence (see item 12).	n/a
Results of individual sources of evidence	17	For each included source of evidence, present the relevant data that were charted that relate to the review questions and objectives.	Tables 4, 5, 7
Synthesis of results	18	Summarize and/or present the charting results as they relate to the review questions and objectives.	12-23
**DISCUSSION**			
Summary of evidence	19	Summarize the main results (including an overview of concepts, themes, and types of evidence available), link to the review questions and objectives, and consider the relevance to key groups.	23-24
Limitations	20	Discuss the limitations of the scoping review process.	24
Conclusions	21	Provide a general interpretation of the results with respect to the review questions and objectives, as well as potential implications and/or next steps.	24-27
**FUNDING**			
Funding	22	Describe sources of funding for the included sources of evidence, as well as sources of funding for the scoping review. Describe the role of the funders of the scoping review.	28

JBI = Joanna Briggs Institute; PRISMA-ScR = Preferred Reporting Items for Systematic reviews and Meta-Analyses extension for Scoping Reviews.
* Where *sources of evidence* (see second footnote) are compiled from, such as bibliographic databases, social media platforms, and Web sites.
† A more inclusive/heterogeneous term used to account for the different types of evidence or data sources (e.g., quantitative and/or qualitative research, expert opinion, and policy documents) that may be eligible in a scoping review as opposed to only studies. This is not to be confused with *information sources* (see first footnote).
‡ The frameworks by Arksey and O'Malley (6) and Levac and colleagues (7) and the JBI guidance (4, 5) refer to the process of data extraction in a scoping review as data charting.
§ The process of systematically examining research evidence to assess its validity, results, and relevance before using it to inform a decision. This term is used for items 12 and 19 instead of "risk of bias" (which is more applicable to systematic reviews of interventions) to include and acknowledge the various sources of evidence that may be used in a scoping review (e.g., quantitative and/or qualitative research, expert opinion, and policy document).

From: Tricco AC, Lillie E, Zarin W, O'Brien KK, Colquhoun H, Levac D, et al. PRISMA Extension for Scoping Reviews (PRISMAScR): Checklist and Explanation. Ann Intern Med. 2018;169:467–473. doi: 10.7326/M18-0850.

For peer review only
